# Phylogeny of the Oniticellini and Onthophagini dung beetles (Scarabaeidae, Scarabaeinae) from morphological evidence

**DOI:** 10.3897/zookeys.579.6183

**Published:** 2016-04-11

**Authors:** T. Keith Philips

**Affiliations:** 1 Systematics and Evolution Laboratory, Department of Biology, Western Kentucky University, Bowling Green, Kentucky 42101, USA; 2 Correspondence: Dr. T. K. Philips, Department of Biology, Western Kentucky University, 1906 College Heights Blvd. #11080, Bowling Green, KY 42101-1080, USA

**Keywords:** Parsimony, Bayesian, biogeography, tribe, subtribe, new taxa

## Abstract

A phylogenetic study was conducted to hypothesize relationships of most of the genera of the Oniticellini and Onthophagini for the first time using morphological characters from a diverse array of external and internal sclerites. The monophyly and sister relationship of both tribes was found using Bayesian and parsimony analyses with heavily to moderately weighted data. An alternative hypothesis based on parsimony analyses of unweighted or slightly weighted data show a paraphyletic Oniticellini without the Onthophagini, although recognition of the subtribe Helictopleurina as a tribe would eliminate non-monophyly.

Of the three Oniticellini subtribes, the Helictopleurina and Drepanocerina are monophyletic. There is no support for the monophyly of the Oniticellina or the Onthophagini subtribe Alloscelina, as currently defined. The genus *Liatongus* is paraphyletic, while strong support was found for monophyly of the Madagascan genus, *Helictopleurus*. The genus *Onthophagus* is never monophyletic in any analysis performed. Two new subtribes are also proposed: Liatongina **subtr. n.** including the genus *Liatongus* and Attavicina **subtr. n.** including the genera Attavicinus and Paroniticellus.

Topological evidence shows that the ancestral oniticellines and onthophagines were all coprophagous with alternative food sources evolving relatively recently. Both myrmecophily and termitophily probably evolved only once in the onthophagines. The phylogenetic analysis supports an African origin for the two tribes, with a relatively early age for the split of the Madagascar helictopleurines from the remaining oniticellines via dispersal. Furthermore, the presence of the oniticellines in the New World is hypothesized to be due to two relatively old dispersal events via Beringia and two relatively recent trans-Atlantic invasions of the Caribbean.

## Introduction

The Oniticellini and Onthophagini contain close to one half of the roughly 7,000 species of scarabaeine dung beetles known worldwide (Schoolmeesters et al. 2010). Undoubtedly more species remain undocumented, perhaps as many as 1,000 or more, and certainly a large proportion of those, when described, will be placed in either the speciose onthophagine genus *Onthophagus* or another closely related group. In one estimate, *Onthophagus* alone contains 1765 species (Hanski and Cambefort 1991) while a recent estimate put the total at ~2500 species (Tarasov and Solodovnikov 2011). With their high species diversity, these two tribes are among the most important in regards to understanding the evolution of the Scarabaeinae dung beetles.

Many Oniticellini and Onthophagini (generic classification in Table 1) are tunnelers where dung is buried at the ends of tunnels created beneath a dropping and used as adult and larval food. But within these tribes the behaviors and food sources are far more diverse. For example, the oniticellines *Oniticellus* Serville, *Tragiscus* Klug, and possibly *Paroniticellus* Balthasar have species with nesting behavior known as dwelling, where brood cavities are excavated within the dung or in the soil adjacent to and in contact with the dropping (Davis 1989, Philips and Bell 2008). Some of the small-sized onthophagines are kleptocoprids that steal dung from larger tunneling or rolling dung beetles. Nearly all oniticellines specialize on dung, especially those from large bodied herbivores (Cambefort 1991). In contrast, many onthophagines have alternative food and nesting habits. This includes taxa that feed on carrion, millipedes, fruit, or mushrooms (Hanski and Cambefort 1991, Brühl and Krell 2003). Several genera are also highly specialized to live with ants or termites and likely feed on nest detritus (Branco 1995, 1996; Krell and Philips 2010). The number of times these alternative food sources have evolved is unknown.

**Table 1.** Generic classification of the Oniticellini and Onthophagini with the number of species in each genus currently recognized in the far right column. Taxa in bold are included in this study.

**Tribe Oniticellini Kolbe, 1905**			
Subtribe Drepanocerina van Lansberge, 1875 – 12 genera, 53 spp. (+ 1 extinct sp.)			
***Afrodrepanus***	Krikken	2009	2
*Clypeodrepanus*	Krikken	2009	3
***Cyptochirus***	Lesne	1900	4
*Drepanocerus*	Kirby	1828	5
*Drepanoplatynus*	Boucomont	1921	1
*Eodrepanus*	Barbero, Palestrini and Roggero	2009	9
*Epidrepanus*	Roggero, Barbero and Palestrini	2015	3
*Ixodina*	Roth	1851	5
*Paraixodina*	Roggero, Barbero and Palestrini	2015	3
*Latodrepanus*	Krikken	2009	3
*Sinodrepanus*	Simonis	1985	8
*Tibiodrepanus*	Krikken	2009	7
Subtribe Oniticellina Kolebe, 1905 – 12 genera, 75 spp.			
***Anoplodrepanus*** *	Simonis	1981	2
***Attavicinus***	Philips and Bell	2008	1
***Euoniticellus***	Janssens	1953	19
***Liatongus***	Reitter	1893	45
*Nitiocellus*	Branco	2010	2
***Oniticellus***	Serville	1828	10
***Paroniticellus***	Balthasar	1963	1
***Scaptocnemis*** **	Péringuey	1901	1
*Scaptodera* #	Hope	1837	1
***Tiniocellus***	Péringuey	1901	6
***Tragiscus***	Klug	1855	1
*Yvescambefortius*	Ochi and Kon	1996	1
Subtribe Helictopleurina Janssens, 1946 – 2 genera and ~ 81 spp.			
***Helictopleurus***	d’Orbigny	1915	62
*Heterosyphus*	Paulian	1975	1
**Tribe Onthophagini Burmeister, 1846 – 36 genera and ~ 2500 spp.**			
Subtribe Alloscelina Janssens, 1949 – 3 genera and 43 spp.			
***Alloscelus***	Boucomont	1923	4
***Haroldius***	Boucomont	1914	38
Megaponerophilus***	Janssens	1949	1
Remaining 33 genera			
***Amietina***	Cambefort	1981	4
*Anoctus*	Sharp	1875	5
***Caccobius***	Thomson	1859	89
*Cambefortius*	Branco	1989	8
***Cassolus***	Sharp	1875	9
***Cleptocaccobius***	Cambefort	1984	24
*Cyobius*	Sharp	1875	5
***Diastellopalpus***	Van Lansberge	1886	33
***Digitonthophagus***	Balthasar	1959	2
*Disphysema*	Harold	1873	1
*Dorbignyolus*	Branco	1989	1
***Euonthophagus***	Balthasar	1959	26
***Eusaproecius***	Branco	1989	7
***Heteroclitopus***	Péringuey	1901	10
Hyalonthophagus***	Palestrini	1989	9
*Krikkenius*	Branco	1989	1
***Milichus***	Péringuey	1901	16
***Mimonthophagus***	Balthasar	1963	8
*Neosaproecius*	Branco	1989	2
***Onthophagus***	Latreille	1802	2000–2500
***Phalops***	Erichson	1847	38
*Pinacopodius*	Branco	1989	2
***Pinacotarsus***	Harold	1875	2
***Proagoderus***	Van Lansberge	1883	73
***Pseudosaproecius***	Balthasar	1941	12
*Stiptocnemis*	Branco	1989	2
***Stiptopodius***	Harold	1871	11
*Stiptotarsus*	Branco	1989	3
Strandius***	Balthasar	1935	17?
Sukelus*	Branco	1992	1
***Tomogonus***	d’Orbigny	1904	11
Unidentis***	Josso and Prévost	2012	1
*Walterantus*	Cambefort	1977	1

* Moved out of the Drepanocerina to the Oniticellina by Krikken (2009).

** Moved out of the Drepanocerina to the Oniticellina by Branco (2010).

# *Scaptodera* is most often cited as Liatongus (Paraliatongus) but is considered a valid genus by some workers.

*** Taxon is considered either a generic synonym or a subgenus by some workers.

The Onthophagini contain approximately 35 genera, the number depending upon the chosen taxonomic classification (Cambefort 1991, Davis et al. 2002, Philips 2011). Many taxa previously considered subgenera are now recognized as genera, including *Proagoderus* Lansberge, *Diastellopalpus* Lansberge, *Digitonthophagus* Balthasar, *Strandius* Balthasar, and *Euonthophagus* Balthasar (Halffter and Matthews 1966, Davis et al. 2008). Subgenera are still recognized in both *Onthophagus* and *Caccobius*, and species groups defined, for example, by Arrow (1931), Balthasar (1963), Boucomont (1923), Branco (1992a) and d’Orbigny (1913) are commonly used. Within the Oniticellini, species groups have been defined, for example, within *Liatongus* and *Helictopleurus* (see Montreuil 2005 for those in the latter). In the Onthophagini, subtribes have yet to be proposed with the exception of the Alloscelina, while three are recognized in the oniticellines. But typically few characters for any of these groups justify their recognition and none have yet been studied in a broader phylogenetic context.

Inconsistencies in the classification of these two tribes have been frequent. For example, nine of the 35 generic level onthophagine taxa were formerly placed in Dichotomiini (Janssens 1939; Balthasar 1941; Branco 1989, 1990, 1991, 1992a, 1992b) or the Oniticellini (Kolbe 1905). The Alloscelina were previously placed as a subtribe of the Scarabaeini (Halffter and Edmonds 1982) or were considered a tribe by Janssens (1949). In a taxonomic proposal for the Drepanocerina (Krikken 2009), the previous species groups of Janssens (1953) were elevated to genera. Most recently, this classification has been updated by Roggero et al. (2015) with the creation of another two genera. Lastly, the monotypic *Scaptodera* (*Scaptodera rhadamistus*) recognized by some workers, has been previously cited as Liatongus (Paraliatongus) Reitter, *Oniticellus* Serville, or even *Pseudoniticellus* Janssens (e.g., Hanski and Cambefort 1991).

Based on evidence of relationships within the Scarabaeinae from a wide range of morphological characters (Zunino 1983, Luzzatto 1994, Philips et al. 2004b, Tarasov and Génier 2015) and some molecular studies (Villalba 2002, Ocampo and Hawks 2006, Monaghan et al. 2007, in part), there is strong support for a single common ancestor for the Oniticellini and Onthophagini. But relationships between and within these two tribes and subtribes are very poorly known. Therefore, this morphological study was undertaken to hypothesize the phylogeny of this group, including a test of monophyly of the entire clade, each tribe, and the four subtribes of the oniticellines and onthophagines currently recognized. Also, a preliminary test of generic monophyly of *Helictopleurus* d’Orbigny, *Liatongus* Reitter and *Onthophagus* Latreille is performed. As is possible with all phylogenies, the evolution of the biology and biogeography of this clade of dung beetles is also hypothesized.

## Materials and methods

The 41 ingroup species, including many rare taxa, represent the morphological and behavioral diversity found within these two tribes reasonably well (Table 2). Representatives of 12 of the 26 genera of Oniticellini were studied, including both Old and New World representatives of *Liatongus* Reitter. Excluded from the study were two monotypic and rarely collected genera *Drepanoplatynus* Kirby, and *Heterosyphus* Paulian, as well as *Sinodrepanus* Simonis. Also, excluded were seven of the recently proposed Drepanocerina genera of Krikken (2009) Barbero et al. (2009a, b), and Roggero et al. (2015) whose species were previously placed in *Drepanocerus*; all of these genera were created after the data collection for this study and their relationships, based on morphological data, have recently been well studied (Roggero et al. 2015). Twenty-four of the 36 genera of Onthophagini were also represented in the data set. Of the 12 excluded genera, all are uncommon in collections and 10 are either monotypic or contain only two species.

**Table 2.** List of taxa and country of collection used in the analysis.

**Ingroup**	
Oniticellini :	
Helictopleurina :	
*Helictopleurus quadripunctatus* Olivier	Madagascar
*Helictopleurus giganteus* Harold	Madagascar
*Helictopleurus neoamplicollis* Krell	Madagascar
Drepanocerina :	
*Scaptocnemis segregis* Péringuey	East Africa
*Cyptochirus ambiguous* (Kirby)	South Africa
*Afrodrepanus impressicollis* (Boheman)	South Africa
Oniticellina :	
*Anoplodrepanus reconditus* (Matthews)	Jamaica
*Tragiscus dimidiatus* Klug	South Africa
*Paroniticellus festivus* Steven	Turkey
*Oniticellus pictus* (Hausmann)	South Africa
*Euoniticellus intermedius* (Reiche)	South Africa
*Liatongus militaris* Castelnau	South Africa
*Liatongus californicus* (Horn)	USA
*Attavicinus monstrosus* (Bates)	Mexico
*Tiniocellus spinipes* Roth	South Africa
Onthophagini :	
Alloscelina :	
*Alloscelus paradoxus* Boucomont	Haut-Uele, Moto, DRC
*Haroldius convexus* (Philips & Scholtz)	South Africa
Subtribe undefined:	
*Amietina eburnea* Cambefort	Ivory Coast
*Caccobius ferrugineus* Fahraeus	Botswana
*Cassolus humeralis* Arrow	Mangpo, Darjeeling, India
*Cleptocaccobius schaedlei* (d’Orbigny)	Kenya
*Diastellopalpus thomsoni* Bates	Malawi
*Digitonthophagus gazella* (Fabricius)	South Africa
*Euonthophagus* sp.	South Africa
*Eusaproecius tinantae* (Boucomont)	DRC
*Heteroclitopus annamariae* Branco	Nigeria
*Hyalonthophagus alcyon* (Klug)	South Africa
*Megaponerophilus megaponerae* Brauns	Lulua, Sandoa, DRC
*Milichus apicalis* Fahraeus	South Africa
*Mimonthophagus hinnulus* (Klug)	Madagascar
*Onthophagus depressus* Harold	South Africa
*Onthophagus hecate* (Panzer)	North America
*Onthophagus capella* Kirby	Australia
*Phalops* sp.	South Africa
*Pinacotarsus dohrni* Harold	Haut-Uele, Paulis, DRC
*Proagoderus lanista* Castelnau	South Africa
*Pseudosaproecius validicornis* Quedenfeldt	Tanzania
*Sukelus jessopi* (Branco)	Kenya
*Stiptopodius doriae* Harold	Gemu-Gofa Prov., Ethiopia
*Strandius lenzi* (Harold)	Japan
*Tomogonus crassus* (d’Orbigny)	Masinga (Mayombe), DRC?
**Outgroups**	
Eurysternini :	
*Eurysternus* sp.	Costa Rica
Sisyphini :	
*Sisyphus* sp.	South Africa
Onitini :	
*Onitis fulgidus* Klug	South Africa

To test generic monophyly, the study included three species of the Madagascar endemic *Helictopleurus* (from three species groups), two species of *Liatongus* Reitter (an Old and New World representative), and three species of *Onthophagus* Latreille (one endemic each from North America, Africa, and Australia). Three outgroup taxa were used to polarize character evolution in the ingroup and included a representative from the Onitini, Eurysternini, and Sisyphini. Previous analyses of the entire subfamily using morphology (Philips et al. 2004b) and evidence from molecular studies (Villalba 2002, Ocampo and Hawks 2006, Monaghan et al. 2007), support the Onitini or a lineage of this tribe (if the Onitini is paraphyletic) as the probable sister clade of the Oniticellini + Onthophagini, and the Sisyphini may also be closely related as well.

Dried specimens were initially relaxed in hot water and then cleared using lactic acid. Individual sclerotized body parts were observed on slides in glycerine and some of the larger structures were studied dry. An attempt was made to discover as many characters as possible from the various sclerotized structures without any bias as to what might be phylogenetically informative. Characters were only excluded if discrete states could not be adequately defined or if they were autapomorphic within a binary character. Five genitalic characters were used and more could have been included (as some recent dung beetle studies have done (e.g., Roggero et al. 2015, Tarasov and Solodovnikov 2011). Regardless, a very broad range and relatively high number (134) of both external and internal morphological characters were discovered (including those from sclerites not used in the previous two studies cited) and used to hypothesize relationships among taxa (see below). Illustrations of nearly all of the character states are in Philips et al. (2002, 2004a, 2004b) and are not duplicated herein. Morphological nomenclature follows primarily Lawrence and Britton (1994), and additionally that of Nel and Scholtz (1990), Doyen (1966) and Dellacasa et al (2010).

For the parsimony analysis, WinClada (Nixon 1999) was used to enter data (Table 3) in Dada, the trees were first produced with Nona (Goloboff 1993a), and the character state distributions were analyzed with Clados (Nixon 2002). Characters were analyzed unordered and initially unweighted. One thousand repetitions with random taxon entry order in Nona were used several times to make certain the shortest trees were discovered, although all of the most parsimonious trees were found within the first 100 replications. Use of the Rachet in TNT (Goloboff et al. 2008) did not find any additional topologies with further searching, using the default parameters as follows: the perturbation phase used up-weighting and down-weighting probabilities of 4 and was stopped when 20 substitutions were made or 99% swapping was completed; the total number of iterations was set at = 10 and the auto-constrained iterations at = 0 with alternate equal weights on.

**Table 3.** Character states (0-133) of the taxa included in the analysis.

Anoplodrepanus
0011121123122102100110101131111101111201011101?201000?????02---2110011111100101000121??11101010101111111111010101120011002110111100211
Cyptochirus
001001113312210210101010123111111111101120111131011101121112-1221100110011101113001211011101010100111101110111111121111002110111100111
Afrodrepanus
00110201421221021011100012311111111110112111113100100112111010221100110111001110001210011101010100111100111110101121111110110111100201
Euoniticellus
02011110531221021001101002311011111110010111110-012001221102---20100111111001113001211111101010111111111110010111121011002110111100211
Helictopleurus_quadripunctatus
001101103312210210111011122111110010121021112110014101121002---21101110110001011010111110001010111110111110010101121011101111111111111
Helictopleurus_giganteus
001000103310210210111011122110110110121021112110012201121102---21101111110001011010111110001010111110111110110101121011101121111111111
Helictopleurus_amplicollis
00110110331221001?111010023102111101101011112131012301121102---21101110110001010011011110001010111110111100111101121011102121111111211
Liatongus_californicus
001001112312210010001010023111111011101111112101011100221102---21100110111011013010211011101010111111111110210101121011102110111101101
Liatongus_militaris
001010113312210011111010023101101011101111112111011101121002---20100110111001010011211000101010001111101110212110121011102110111101211
Liatongus_monstrosus
001010111311210210111010023112132011121121112111011101121002---21101111111102013000210011001010111110111110113111021111102110111111211
Oniticellus
001011112312210210201010023111101111100101112132011101121102---20101110111011110001111100101010011111101110311110121001002110111111211
Paroniticellus
001010112312210210111010023110110011101021111131011401121102---21101110010101011001210001101010001111101110110111021111102110111111111
Scaptocnemis
00101011311221021001101002311011001110110011113201100022??12-022110011011001111301121111110101111110110111011211112101100211011110121?
Tiniocellus
0011121123122102101010101131101?0011101100112132011001121102---20111010110001111011211101101010011101101110011101121011002110111101211
Tragiscus
0010?00101112102100010100231111?111110110111210-011211121002---1110111011101111300121101110101011111110111031211002100101211011111121?
Haroldius
00111220731200011?11101011311211110102112?11013211060012??101020111110111?320013211211110111210111111011111412001021211601102101111011
Alloscelus
111011106012010210301112123112101111111101110132103001121012-022110111111?020003111111111110000111111110111112111000111201106001110010
Amietina
01111200331221001030101001301110110102112211013210300?????12-020111111011100001311101??11111010111111110111112111100111241106011111110
Caccobius
02111210731221021010101002310010010112112011211110?020121012-1221100111110002013010211011001010111110110101112111100111301100111111111
Cassolus
0000021002122002001013101031121111010211221131?211053?????101020111111111102011311101??10101310111011011111112011001111641105001111110
Cleptocaccobius
021112007312210010101000013111100121121120110132105000221111-0201110111010002013011111111101010111111110101112111100111201100111111111
Diastellopalpus
001013101210210210111010020113000121121111012111014101121112-201110111110100101301021101100101011111111010111211111011150100311111111?
Digitonthophagus
100010102112210000011010113110120111121111102131013201121112-1211101111110101013010201011001010111111111101112111110011301100111111111
Euonthophagus
001112103212210210411010023100110101121101102131003101121012-022110111011000201201111111010101011111111011111211110011130110011111111?
Eusaproecius
021112103012110211410010101112131101111111110032103121121012-1221001101110020013001211111121200111111110111112111100111201100111111111
Heteroclitopus
021112103002111210?1100010311211?111111101110032103321121012-0221111101110?20003001011110121201111111110111112111100111201100110011011
Hyalonthophagus
121002107112210210011010023100100121121111012131003400221012-0021001110010002013010011111101010110110110111112111100110701100111111111
Megaponerophilus
03111200?312010110101000113101100111011101112031103401121012-1201111101110221113111211111111200111111110111112111000111701106101111110
Milichus
031112207312210200211010023001100121121101102131003021121012-1201101111010002013000211011101010111110110111112111110111341100111111111
Onthophagus_capella
121101106210210210501111123112110111121111102131016100121012-0221101111001002013010111010101010111110110101102111110111301100111111111
Onthophagus_depressus
000111108312210010011010113112010111121111100131003201121112-0221111110110102013000011011101010111111110101102111100111201100111110011
Onthophagus_hecate
021112107212210010211010113111110111121111104131004400221012-1221001110111032010010011011001010111110110101112111100111201100111111111
Onthophagus_sp.
020112204312210200101010013100100111121101112121103100221112-0211111011110102013011011011101010011111110101102111100111301100111111111
Phalops
001002112112210200011010113111110100121111104011011201121012-0011001110110101010000211011101010111111111101112111121110341100111011111
Pinacotarsus
0211121030121102013100121011121421111111?1110032103101121012-0201111101110000003001011111121100111111111111111111100111401100110111011
Proagoderus
00100210231221021051101002311112010112110111211-016101121112-1211001110010001010011011011001010111110011111112111001111501100111111111
Pseudosaproecius
0211121070120102000101101031121?110112111111013110?00112101210221101111110320?13011011011110200111111111111112111100111301100111111011
Sukelus
021112101002211211310012101112152111111111100032103100121012-020110110111???0?03011211111121200111111110111112111100111201100110011011
Stiptopodius
0211111030121112013102121011121?2101121121110032103021121012-0221111101110020013001111111121100111111111111112111100111201100101111011
Strandius
121111108312210200411110113111100111121111102130004100211012-0021101110010002012010211010101010111110111111113111110111301100111111111
Tomogonus
1111111093122000101011101130111001211211111021?110300?????12-122110111111002201301121??1110101011121011111111?111100111301106111111011
Eurysternus
1001121003122101021112100131041?01010211201121320055302101100112110111111030001311101111110101111111111101051011113001183011601111011?
Onitis
002001110312210213111110023114100121021121112110016200000112-0221101111110001013000201111001010111011111110512111030011820014111111111
Sisyphus
03111210731221021011112012311210011102112211113200220000011001121101110110000013100011111101011111111111011014001130011951106011110101

Piwe weighting (parsimony with implied weighting) in TNT (Goloboff 1997) was used in an attempt to reduce the number of equally parsimonious trees. K values were tested from 1 through 50 using the default parameters. This program estimates character weights (i.e., character reliability based on degree of homoplasy) during the tree search and is based on searching for trees which have maximum total fit. The algorithm considers the fit as a concave function of homoplasy and, when comparing topologies, differences in steps occurring in characters which show more homoplasy, are less influential. In other words, if two trees of equal length are being compared, the tree topology supported by the character with less homoplasy is preferred (see Goloboff 1993b for more details). Analyses with lower K (or CO) values have the strongest weighting against those characters with homoplasy.

Weighting in morphological analyses has been considered unscientific by some (e.g., Kluge 1997) but valid by many others (e.g., Griswold et al. 1999, Platnick 2000). It could be argued that giving all characters equal weights is a form of unbiased weighting. Certainly some characters are more informative than others in any analysis and differential weighting is considered a reasonable technique to use in the present study (see Goloboff et al. 2008 for further discussion and citations within).

The standard model for morphological data and the default set of priors were used in the Bayesian analysis (Mr. Bayes version 3.2.1 for Windows 32 bit) (Ronquist et al. 2012). The topology was developed using the MCMC command with two simultaneous searches. Three separate runs were performed and for each 1,000,000 generations were typically needed to get below the 0.01 level of the standard deviation of split frequencies. Default burn-in values used were the first 25% from the cold chain. Plots of the likelihoods of sampled trees were examined to determine when the MCMC chains had reached the stationary distribution. The majority rule consensus tree was obtained from the remaining trees.

Posterior probability node support is shown on the tree from the Bayesian analysis. Bootstrap values were calculated in TNT from 1000 replicates using sampling with replacement, the traditional tree search setting, and collapsing of groups below a value of 1. Values are displayed at all nodes where support is at or above 0.5 or 50%. Consistency and retention indices (CI and RI) derived from unweighted data (Figs 4–5) are listed after each character within the descriptions. CI and RI values for weighted data (not shown) would result in different values for many characters depending upon the K value used; additionally the lower the K value the more likely characters would receive values of zero.

Biogeographic analysis was done using a Dispersal-Vicariance Analysis (Ronquist 1997) in S-DIVA (Yu et al. 2010) to hypothesize ancestral state distributions with the maximum areas at each node = 4 to reduce erroneously inferred vicariance events. The included areas were Afrotropical, Palaearctic, Oriental, Australasian, Madagascar, Nearctic, Neotropical, and Caribbean following those recognised for dung beetles in Davis et al. (2002).

Brief discussions on the evolution of morphology, nesting behavior, food sources, and biogeography are based on the best supported hypotheses of evolution. Rather than inclusion in the discussion, Table 4 is used to summarize the specific character support based on unweighted and/or weighted K = 10 topologies for the tribes, subtribes, and relevant clades. One should note that weighted tree support is based on synapomorphies that can be considered more reliable in defining clades.

**Table 4.** Character support for selected clades, unweighted or weighted (K value =10) data.

**Character support: Onitini, Oniticellini, and the Onthophagini monophyly (unweighted data)**
**Uncontroverted synapomorphies**:
1) pygidium median groove absent (59-2);
2) propygidium transversely with an even width (61-0);
3) propygidium posterior/ventral border medially without either an angulate emargination anteriorly or rounded shape posteriorly (62-2);
4) elytral striae composed of a single line (94-0);
5) cervical lateral sclerite apex lacking a lateral pocket or cavity (104-1);
6) meso- and metatibia broad and greatly expanded apically (125-1);
7) metatibia not elongate and parallel sided at middle ½ (130-1).
**Controverted synapomorphies**:
8) cephalic projection consisting of a central horn positioned posteriorly (46-1);
9) eye canthus completely dividing eye (49-1);
10) ventral lacinial articulation sclerite with a short “tail” extending only part of length (76-1);
11) four teeth on the protibia; (80-0);
12) pronotal posterior margin with a slight but distinct posteriorly directed point or angulate edge (89-0).
**Character support: Oniticellini + Onthophagini monophyly (unweighted data)**
**Uncontroverted synapomorphies**:
1) labial palps with the third palpomere reduced in size and appearing as having only two palpomeres (21-0);
2) mentum paraglossal strut laterally at distal apex expanded but not bifurcate (29-1);
3) angle of parameres to basal piece where attached approximately 45 degrees (54-1);
4) length of parameres to basal piece, excluding narrow projections approximately ½ the length of basal piece (55-2);
5) dorsal plate of the genital capsule oriented transversely (56-1).
**Controverted synapomorphies**:
1) mentum glossal lobe narrowly rounded (31-1);
2) the antennae with vessicles present and easily visible (36-1);
3) blunt paramere apex (53-1);
4) mesonotum with the prescutum anteriorly transverse plate approximately ventrally directed and angularly emarginate (107-1);
5) mesocutum lateral projection extended with a pointed apex (114-2);
6) mesoscutum lateral edge lacking a pocket (115-1);
7) metascutellum posterior edge with a slight margin or remnant edge (119-1).
**Character support: Oniticellini + Onthophagini monophyly (weighted data, k = 10)**
**Uncontroverted synapomorphies**:
1) labial palps with the third palpomere reduced in size and appearing as having only two palpomeres (21-0);
2) angle of parameres to basal piece where attached approximately 45 degrees (54-1);
3) length of parameres to basal piece, excluding narrow projections approximately ½ the length of basal piece (55-2);
4) dorsal plate of the genital capsule oriented transversely (56-1);
5) wing AA vein posteriorly forming a cell partially closed with extension toward costal margin (8-2).
**Controverted synapomorphies**:
1) antennae with vessicles present and easily visible (36-1);
2) blunt paramere apex (53-1);
3) mesonotum with the prescutum anteriorly transverse plate approximately ventrally directed and angularly emarginate (107-1);
4) paraglossal strut laterally at distal apex expanded, but not bifurcate (29-1);
posterior margin of the gula on the head truncate and slightly projecting (51-1).
**Character support: Oniticellini monophyly (weighted data, k = 10)**
**Uncontroverted synapomorphies**:
1) pygidium lacks a transverse ridge defining or separating the propygidium from the pygidium (58-0).
**Character support: Onthophagini monophyly (unweighted data)**
**Uncontroverted synapomorphies**:
1) scutellum (metanotum) posterior margin with a short apical projection and with a truncate apex and laterally with rapidly converging sides (119-5).
**Controverted synapomorphies**:
1) dorso-lateral edge of propygidium angulate and not rounded; (63-1);
2) mesonotum scutellum apex triangular shaped (106-1);
3) prescutum-scutum junction when viewed in a horizontal position broadly rounded (109-2);
4) mediophragma of the metanotum with a distinct longitudinal ridge (123-0).
**Character support: Onthophagini monophyly (weighted data, k = 10)**
**Controverted synapomorphies**:
1) dorso-lateral edge of propygidium angulate, and not rounded (63-1);
2) the scutellum (mesonotum) apex triangular shaped (106-1);
3) prephragma of the prescutum (mesonotum) in ventral view recurved in shape (116-1);
4) mediophragma (metanotum) with a distinct longitudinal ridge (123-0).
**Onthophagini clade 1 support: *Haroldius + Cassolus* through to *Sukulus* (unweighted data)**
**Uncontroverted synapomorphy**:
1) apical extension of pigmented mesal comb compared to sclerotized (darkened) area near opposite lateral edge approximately the same length or slightly shorter; (92-2).
**Controverted synapomorphies**:
1) mentum length approximately equal to width (24-1);
2) inner strut of lacinia (nearest to palpifer), distal tip, notch absent and tapered tip (75-2);
3) scutum distinctly transverse (113-0);
4) metatarsi: length of first metatarsis compared to second: first nearly 2X or more the length of second (126-0).
**Onthophagini clade 2 support: *Megaponerophilus* through to *Sukulus* (unweighted data)**
**Uncontroverted synapomorphies**:
1) proximal antennomere of the apical club in lateral view (~4 to 5 times as long as wide) (37-1);
2) mandibular cuticle on the lateral edge and part of ventral surface bright chestnut red color (93-0).
**Controverted synapomorphies**:
1) prothoracic apodemes with a complete oblique suture (40-0);
2) apex of the paramere blunt and not pointed (53-1).
**Oniticellini clade 1 support: Helictopleurina (unweighted data)**
**Controverted synapomorphies**:
1) prothoracic apodeme with a large flattened single flange and with one lateral edge slightly expanded perpendicularly (39-0) [only seen elsewhere in *Paroniticellus*];
2) pronotal surface glabrous (88-0);
3) elytral seventh stria strongly curved (100-0);
4) anterior margin of scutum with an pale colored, transverse region of cuticle (111-0).
**Oniticellini clade 2 support: Drepanocerina: *Cyptochirus + Afrodrepanus* (unweighted data and weighted data, k = 10)**
**Controverted synapomorphies**:
1) anal region of wing with a posterior notch (4-0);
2) apodeme of cervical sclerite lacking a carina (40-2);
3) a single carinae anterior of eyes (47-1);
4) a transverse ridge defining or separating propygidium from the pygidium present (58-1);
5) Elytral umeral angle in dorsal view with a lateral bulge (97-0);
6) Mesonotum prescutum: prephragma in ventral view with recurve present (116-1).
**Oniticellini clade 3 support: Oniticellina and Drepanocerina (unweighted data)**
**Controverted synapomorphies**:
1) RA vein extension along apical margin relatively great (7-1) [only seen elsewhere in *Phalops* and *Onitis*];
2) internal apodeme (V or Y shaped usually with one arm relatively short) adjacent to paraglossal apodeme in dorsal view, from anterior to posterior obliquely angled outwards (33-0) [only seen elsewhere in *Helictopleurus quadripunctatus*].
**Oniticellini clade 4 support: *Attavicinus* + *Paroniticellus* (unweighted data)**
**Controverted synapomorphies**:
1) internal accessory sclerite within the maxilla that is curved near the proximal end (74-1);
2) the protibia on proximal side with a line of setae associated with a carina (81-0);
3) males with two postero-lateral ridges on the pronotum (85-0);
4) scutum distinctly transverse (113-0).
**Oniticellini clade 5 support: *Liatongus californicus* to *Euoniticellus* (unweighted data)**
**Uncontroverted synapomorphies**:
1) metacoxal separation at middle relatively large (129, 0), uncontroverted.
**Controverted synapomorphies**:
2) labium internal apodeme adjacent to paraglossal apodeme in dorsal view, from anterior to posterior, appearing V shaped (32, 1);
3) visible portion of the first ventrite projecting between metacoxae distinctly truncate at apex (67, 0).

## Description of the morphological characters and their states

### Wing

0. AA vein: (0) with a remnant cross vein and appearing as a small “T” proximally; (1) lacking remnant cross vein. CI = 0.16, RI = 0.16.

1. MP vein length compared to RP: (0) normal (long), approximately 30% or more the length of the RP; (1) intermediate, approximately 20–30% the RP length; (2) short, only near margin and not extending backwards (less than 20% the RP length); (3) MP vein absent. CI = 0.30, RI = 0.58.

2. Pigment patch near apical 1/3: (0) absent; (1) present and distal edge perpendicular to longitudinal axis of the body (Note that the patch is sometimes poorly defined; (2) present and distal edge oblique. CI = 0.28, RI = 0.0.

3. Anal region: (0) large and well developed (noticeably expanded); (1) lacking this shape. CI = 0.10, RI = 0.43.

4. Anal region posterior notch: (0) present; (1) absent. CI = 0.11, RI = 0.27.

5. Jugal and AP vein: (0) both present; (1) AP only; (2) both absent; (3) jugal only. CI = 0.17, RI = 0.22.

6. MP vein near posterior margin of wing: (0) straight to very slightly curved; (1) distinctly, strongly curved; (2) does not reach margin. CI = 0.20, RI = 0.0.

7. RA vein extension along apical margin: (0) slight, not reaching the margin; (1) great, reaching and running parallel to margin for part of its length. CI = 0.25, RI = 0.75.

8. AA vein posteriorly (near wing base): bridge vein extension towards CuA vein: (0) forming cell that is completely closed; (1) forming cell partially closed and extending on both sides of AA and AA vein with small break; (2) forming cell partially closed and with extension toward costal margin but large break on AA vein; (3) cell broadly open, bridge vein absent; (4) forming cell slightly closed with bend towards costal margin; (5) forming cell slightly closed and proximal end of bridge vein detached from AA vein; (6) cell bridge vein complete but cell partially open due to break in AA vein; (7) cell absent (lacking bridge vein) and AA vein smoothly curved; (8) cell partially closed with bridge vein not reaching CuA vein; (9) cell absent (bridge vein absent) with vein slightly sigmodal in shape. CI = 0.39, RI = 0.36.

### Epipharynx

9. Apical margin: (0) broadly emarginate and not projecting medially; (1) narrowly, smoothly emarginate medially; (2) projecting medially; (3) approximately truncate. CI = 0.25, RI = 0.40.

10. Pigmentation: (0) extremely heavy and dark colored; (1) heavy and dark color absent. CI = 0.50, RI = 0.0.

11. Lateral comb (chaetopariae) apical extension: (0) to or near lateral margin; (1) to front margin first and curving towards lateral margin; (2) distant from both front and lateral margin. CI = 0.40, RI = 0.0.

12. Lateral combs (chaetopariae): (0) relatively parallel and straight but curving slightly inwards apically; (1) relatively parallel and straight throughout; (2) curved throughout. CI = 0.50, RI = 0.66.

13. Anterior median ventral process base (crepis): (0) long (length much longer than width; (1) short (length approximately equal to width). CI = 0.50, RI = 0.50.

14. Anterior median ventral process basally (crepis): (0) distinct and triangular (sometimes elongately); (1) indistinct. CI = 0.50, RI = 0.50.

15. Posterior median tormal process (epitorma): (0) smoothly, broadly tapered throughout: (1) smoothly narrowly tapered; (2) more irregularly shaped. CI = 0.18, RI = 0.10.

16. Lateral tormal processes (apotorma): (0) without any obvious circular-shaped muscle attachment point proximally; (1) with attachment point(s). CI = 0.10, RI = 0.0.

17. Dark pattern on anterior half of ventral surface: (0) broadly triangular without any emargination on either side of peak; (1) moderately broadly triangular and with an emargination on either side of the peak; (2) triangular but with parallel sides at narrow base; (3) broadly rounded. CI = 0.60, RI = 0.50.

18. Cavity on dorsal side of proximal portion of epipharynx: (0) broadly emarginate; (1) flat to slightly rounded; (2) narrowly, sharply emarginate; (3) narrowly, gradually emarginate; (4) moderately, sharply emarginate; (5) small shallow emargination on either side of middle (two). CI = 0.27, RI = 0.27.

19. Base of anterior median process in dorsal view (crepis): (0) lateral projections visible; (1) projections lacking. CI = 0.11, RI = 0.38.

20. Coarse setae on ventral surface between combs (prophobae): (0) at middle only; (1) relatively evenly scattered throughout. CI = 0.50, RI = 0.75.

### Labium

21. Third palpomere: (0) indistinctly and appearing as two, with the third very reduced in size; (1) distinctly as three, third only slightly reduced; (2)third absent; (3) third subequal to second. CI = 0.37, RI = 0.28. Note that in state “0,” the third palpomere is very tiny and difficult to observe. It appears though to be a tiny reduced palpomere.

22. Second palpomere shape: (0) distinctly rounded to transverse; (1) elongate; (2) triangular. CI = 0.40, RI = 0.0.

23. Mentum apical edge: (0) shallow V-shaped notch; (1) U-shaped notch; (2) smoothly rounded notch. CI = 0.40, RI = 0.40.

24. Mentum shape: (0) distinctly transverse; (1) length approximately equal to width. CI = 0.10, RI = 0.52.

25. Mentum oblique lateral edge: (0) no notch, steeply angled; (1) no notch, moderately angled; (2) notch present (slight to distinct). CI = 0.18, RI = 0.52.

26. Shape of anterior declivous region of mentum: (0) narrow and parallel sided; (1) elongate triangular shape with acutely pointed apex; (2) broad and parallel sided; (3) broad triangular shape with a broadly rounded apex. CI = 0.75, RI = 0.75.

27. Anterior declivous region of mentum apex: (0) with a dark lip; (1) dark lip absent. CI = 0.33, RI = 0.0.

28. Anterior declivous region of mentum: (0) projecting anteriorly with ventral surface horizontal; (1) or not. CI = 0.12, RI = 0.12.

29. Paraglossal strut laterally at distal apex: (0) expanded and bifurcate; (1) expanded but not bifurcate; (2) approximately parallel sided; (3) sigmoidal; (4) abruptly expanded near apex. CI = 0.22, RI = 0.39.

30. Palpomere strut apex adjacent to paraglossal strut: (0) expanded; (1) unexpanded. CI = 0.50, RI = 0.0.

31. Glossal lobe; (0) large and emarginate; (1) large and narrowly rounded; (2) large and broadly rounded; (3) large and triangular; (4) small and rounded; (5) absent. CI = 0.33, RI = 0.37.

32. Internal apodeme (V or Y shaped usually with one arm relatively short) adjacent to paraglossal apodeme in dorsal view, from anterior to posterior, appearing: (0) Y-shaped; (1) V-shaped; (2) straight, no arm present. CI = 0.25, RI = 0.62.

33. Internal apodeme (as in #32): (0) obliquely angled outwards; (1) longitudinally aligned; (2) obliquely angled inwards. CI = 0.33, RI = 0.66.

34. Internal apodeme, proximal apex: (0) enlarged; (1) not enlarged. CI = 0.12, RI = 0.12.

35. Most proximal apodeme, length from apex to transverse bridge: (0) long; (1) normal (approximately the same length as the width of the transverse bridge). CI = 0.50, RI = 0.50.

### Antennae

36. Antennal vessicles: (0) absent (or possibly obscure and difficult to see); (1) present and easily visible. CI = 0.33, RI = 0.66.

37. Proximal antennomere of the apical club viewed laterally: (0) about 50% longer than wide; (1) approximately 4 to 5 times as long as wide; (2) approximately two times as long as wide. CI = 0.28, RI = 0.66.

### Prothoracic apodemes

38. Apical flanges: (0) trilobed; (1) or not. CI = 0.50, RI = 0.50.

39. Apodeme: (0) with a large flattened single flange (with one lateral edge slightly expanded perpendicularly; (1) large flattened single flange absent. CI = 0.50, RI = 0.66.

40. Apodeme: (0) with a complete oblique suture/carina; (1) with an incomplete oblique suture/carina; (2) suture/carina absent. CI = 0.14, RI = 0.53.

41. Apodeme pocket: (0) deep and elongate; (1) no deep pocket, slight flanges at most. CI = 0.25, RI = 0.14.

42. Apodeme apex: (0) with a short second flange; (1) short second flange absent. CI = 0.50, RI = 0.0.

43. Apodeme shape: (0) broad, smoothly rounded; (1) lacking a broad and smoothly rounded shape. CI = 0.25, RI = 0.66.

### Head

44. Clypeal teeth: (0) two and juxtaposed; (1) two and broadly separated; (2) four; (3) single median. CI = 0.36, RI = 0.58.

45. Genal development: (0) strong, acutely angled or less; (1) weak, angle greater than 90 degrees. CI = 0.33, RI = 0.66.

46. Cephalic projections: (0) central horn anteriorly; (1) central horn posteriorly; (2) paired separate projections. CI = 0.30, RI = 0.30.

47. Carinae anterior of eyes: (0) two transverse carinae; (1) one transverse carina; (2) no carinae. CI = 0.22, RI = 0.61. Taxa that have a horn that obscures a carina if it was present were coded with a dash (-).

48. Transverse projection: horn or ridge posterior of eyes: (0) present; (1) absent. CI = 0.50, RI = 0.92. This ridge is located anterior of a second ridge, if present, demarking smooth usually hidden surface posterior from typically exposed part of the head.

49. Eye canthus: (0) not dividing eye; (1) completely dividing eye. CI = 0.20, RI = 0.80.

50. Head posterior margin, dorsally: (0) wide projection with broadly truncate apex; (1) medial moderate triangular projection approximately equal in size to emarginations on either side; (2) medial narrow triangular projection, much narrower than emarginations on either side; (3) medial broad triangular projection, larger than emarginations on either side; (5) medial broad and elongate triangular projection and with emarginations distally with slightly filled in with darken cuticle; (6) medial very broad projection with approximately equal and broad emarginations on either side. CI = 0.42, RI = 0.61.

51. Posterior margin of gula: (0) broadly rounded and strongly projecting; (1) truncate and slightly projecting; (2) shallowly emarginate at middle; (3) narrowly slightly projecting at middle; (4) broadly rounded and weakly projecting; (5) truncate but narrowly emarginate at middle; (6) narrowly strongly projecting at middle. CI = 0.27, RI = 0.30.

52. Setal pattern on anterior margin of gula: (0) transverse band; (1) elongate triangular shape with base positioned distally; (2) approximately equilateral triangle with base positioned proximally; (3) broad triangular shape with base positioned distally. CI = 0.37, RI = 0.0.

### Aedeagus and genital capsule

53. Paramere apex: (0) elongately pointed; (1) blunt. CI = 0.12, RI = 0.46.

54. Angle of parameres to basal piece where attached: (0) aligned at approximately same angle; (1) approximately 45 degrees; (2) approximately 90 degrees. CI = 0.20, RI = 0.11.

55. Length of parameres to basal piece, excluding narrow projections: (0) nearly equal to length of basal piece; (1) much greater than ½ the length of basal piece; (2) approximately ½ the length of basal piece. CI = 0.66, RI = 0.50.

56. Dorsal plate orientation: (0) vertical; (1) transverse. CI = 1.00, RI = 1.00.

57. Dorsal plate shape: (0) with distinct lateral projections or “tails;” (1) lateral projections lacking. CI = 0.12, RI = 0.58.

### Pygidium

58. Transverse ridge defining or separating propygidium from the pygidium: (0) absent; (1) present. CI = 0.25, RI = 0.72.

59. Median groove: (0) distinct and with complete lateral edges; (1) present but indistinct; (2) absent. CI = 0.50, RI = 0.50.

60. Median groove on propygidium: (0) sides parallel; (1) sides converging. CI = 1.00, RI = 1.00.

61. Propygidium shape: (0) even width throughout; (1) longer medially (slightly); (2) narrowed medially. CI = 0.18, RI = 0.10.

62. Propygidium posterior/ventral border medially: (0) angulate emargination anteriorly; (1) rounded posteriorly; (2) none of the previous. CI = 0.40, RI = 0.25.

63. Dorso-lateral edge of propygidium: (0) with a lateral projection creating an overhanging edge; (1) not projecting and angulate, not rounded; (2) none of the previous. CI = 0.22, RI = 0.41.

64. Dark band of pigment at base: (0) present; (1) absent. CI = 0.25, RI = 0.0.

65. Propygidium length compared to width: (0) relatively broad; (1) or narrow. CI = 0.20, RI = 0.0.

### Abdominal ventrites

66. First ventrite: (0) distinctly visible throughout entire length; (1) reduced to thin “line” near middle. CI = 0.16, RI = 0.50.

67. First ventrite visible portion projecting between metacoxae: (0) distinctly truncate; (1) not distinctly truncate. CI = 0.20, RI = 0.50.

68. Fifth ventrite, males: (0) reduced to a thin line at middle; (1) wider and obvious. CI = 0.50, RI = 0.0. Note: Males were lacking for *Liatongus californicus*, *Anoplophora*, *Cassolus*, *Amietina*, and *Tomogonus* and were coded as “?.”

### Maxillae

69. Galea shape: (0) longer than wide; (1) wider than long. CI = 0.33, RI = 0.66.

70. Apical palpomere: (0) basal 2/3 distinctly darker than apical 1/3; (1) approximately the same shade throughout. CI = 0.80, RI = 0.42.

71. Dorsal articulatory sclerite: (0) projecting laterally as an acute point; (1) not projecting laterally as an acute point. CI = 0.14, RI = 0.14.

72. Ventral articulatory sclerite (‘V) of the galea (distal half) in dorsal view: (0) abruptly expanded from base to apex on galea brush side at middle; (1) expansion not abrupt but more gradual. CI = 0.50, RI = 0.0.

73. Ventral articulatory sclerite (‘V’) of the galea (proximal half) in dorsal view: (0) parallel sided or nearly so; (1) expanded at or near middle. CI = 0.12, RI = 0.46.

74. Accessory internal sclerite: (0) curved near distal end; (1) curved near proximal end; (2) straight. CI = 0.33, RI = 0.25. Two taxa, *Heteroclitopus* and *Sukelus*, were coded as “?” as the structure was too difficult to see clearly.

75. Inner strut of lacinia (nearest to palpifer), distal tip: (0) with notch (sometimes with one tooth greatly reduced); (1) with notch and with one tooth perpendicular to other; (2) notch absent and tapered tip; (3) notch absent and spatulate tip. CI = 0.42, RI = 0.63.

76. Ventral lacinial articulation sclerite (in ventral view): (0) lacking any extension or “tail;” (1) with a short “tail” extending only part of length; (2) with a long “tail” extending most of lacinial length. CI = 0.40, RI = 0.86.

77. Number of “extra” sclerites associated with the galea: (0) one or more; (1) absent. CI = 0.25, RI = 0.62.

78. Large sclerotized plate of the dorsal aticulation sclerite situated between arms of ventral articulatory sclerite (“V) of the galea: (0) present; (1) absent. CI = 0.33, RI = 0.33.

79. Ventral articulatory sclerite (‘V) of the galea, apex of distal arm: (0) truncate; (1) slightly emarginate; (2) bifurcate; (3) pointed or rounded. CI = 0.23, RI = 0.09.

### Pronota and prolegs

80. Number of teeth on tibia: (0) four; (1) three; (2) two. CI = 0.50, RI = 0.60.

81. Tibia on proximal side opposite outer teeth: (0) with a line of setae associated with a carina; (1) setae absent. CI = 0.11, RI = 0.50.

82. Pronotum anterior opening dorsally: (0) with a slight indentation medially; (1) or more smoothly rounded. CI = 0.12, RI = 0.53.

83. Tibia between second and third teeth, number of microteeth: (0) two or three; (1) one; (2) none. CI = 0.11, RI = 0.15.

84. Male tibia: (0) elongate, narrow, and curved near apex; (1) relatively short, wide and straight throughout. CI = 0.50, RI = 0.0.

85. Male pronotal sculpture: (0) two posterio-lateral ridges; (1) or not. CI = 0.50, RI = 0.50.

86. Male pronotal projections or depressions: (0) present; (1) absent, surface smoothly rounded. CI = 0.12, RI = 0.61.

87. Pronotum with pale colored maculations: (0) present; (1) absent. CI = 0.25, RI = 0.0.

88. Pronotal surface: (0) glabrous; (1) setose, although sometimes only at or near edges. CI = 0.14, RI = 0.40.

89. Pronotum posterior margin dorsally: (0) with a slight but distinct posteriorly directed point or angulate edge; (1) edge rounded. CI = 0.14, RI = 0.33.

### Mandibles

90. Apex, appearing (0) sharply acute; (1) narrowly rounded; (2) broadly rounded. CI = 0.66, RI = 0.87.

91. Outer lateral edge: (0) bulbous/expanded laterally; (1) no bulbous expansion laterally. CI = 0.50, RI = 0.0.

92. Apical extension of pigmented mesal comb compared to sclerotized (darkened) area near opposite lateral edge: (0) longer; (1) absent (not visible); (2) approximately the same length or slightly shorter; (3) approximately ¾ the length of the sclerotized area. CI = 0.60, RI = 0.66.

93. Outer lateral edge and part of ventral surface: (0) with bright chestnut red colored cuticle; (1) red colored cuticle absent. CI = 1.00, RI = 1.00.

### Elytra

94. Elytral striae: (0) composed of a single line; (1) composed of a three lines creating two rows (i.e., with a thin line separating two adjacent rows). CI = 0.33, RI = 0.33

95. Pigment patches: (0) dark elongate areas on some intervals adjacent to pale cuticle; (1) dark areas lacking. CI = 0.20, RI = 0.0.

96. First, third and fifth intervals: (0) slightly raised above surrounding intervals; (1) nearly the same height as the others. CI = 0.25, RI = 0.25.

97. Humeral angle in dorsal view: (0) with a lateral bulge; (1) bulge absent. CI = 0.50, RI = 0.50.

98. Epipleura: (0) double; (1) single; (2) indistinct. CI = 0.66, RI = 0.0.

Note in *Tomogonus* there is no distinct epipleura but the elytra are smoothly rounded to the lateral edge.

99. Eighth interval: (0) extending only approximately ½ the length of the elytron; (1) or not. CI = 1.00, RI = 1.00.

Note that in *Scaptocnemis* and *Tiniocellus*, the eight interval appears to be obsolete beyond the basal half.

100. Seventh stria: (0) strongly and distinctly curved inwards towards 6^th^ stria near the elytral base; (1) slightly curved at most. CI = 0.16, RI = 0.54.

### Cervical lateral sclerite

101. Shape at anterior margin: (0) rounded without or only a slight lateral protrusion; (1) or not. CI = 0.50, RI = 0.50.

102. Lateral margin near apex: (0) with a distinct beak-like tapered straight point; (1) tapered straight point absent. CI = 0.33, RI = 0.71.

103. Apex: (0) with a distinct darkened hook projecting ventrally; (1) hook projecting ventrally absent. CI = 0.11, RI = 0.50.

104. Apex with a lateral pocket or cavity: (0) present; (1) absent. CI = 1.00, RI = 1.00.

105. Apex, including muscle apodeme: (0) broadly rounded; (1) or not. CI = 0.16, RI = 0.44.

### Mesonotum

106. Scutellum apex: (0) elongate and parallel sided, tongue-shaped; (1) triangular shaped. CI = 0.25, RI = 0.78.

107. Prescutum anteriorly: transverse plate approximately ventrally directed: (0) narrow, parallel sided, needle-shaped; (1) angularly emarginate to various depths; (2) narrowly rounded; (3) short small projection; (4) flat, no projection; (5) broadly rounded. CI = 0.55, RI = 0.42.

108. Prescutum anteriorly transverse plate approximately ventrally directed: (0) with distal tips curved out laterally; (1) tips parallel or absent. CI = 0.33, RI = 0.0.

109. Prescutum-scutum junction when viewed in a horizontal position: (0) sharply emarginate; (1) broadly emarginate; (2) broadly rounded; (3) narrowly rounded at middle; (4) straight. CI = 0.33, RI = 0.33.

110. Scutum- scutellum, each sclerite: (0) on two different planes or levels (with a strong declivity between them); (1) on a single plane or level (declivity absent or weak). CI = 0.50, RI = 0.50.

111. Anterior margin of scutum: (0) with a pale colored, transverse region of cuticle; (1) pale colored transverse region absent. CI = 0.14, RI = 0.25.

112. Prescutum: prephragma (longitudinally directed median apodeme with scutum in a horizontal position: (0) with a transverse suture; (1) transverse suture absent. CI = 0.50, RI = 0.50.

113. Scutum: (0) distinctly transverse; (1) not distinctly transverse in shape. CI = 0.16, RI = 0.37.

114. Scutum lateral projection (0) extended only slightly and truncate; (1) distinctly extended and with a blunt apex; (2) extended and with a pointed apex; (3) projection absent. CI = 0.37, RI = 0.77.

115. Scutum lateral edge: (0) with a rounded or somewhat rounded lateral pocket; (1) pocket absent. CI = 0.20, RI = 0.76.

116. Prescutum: prephragma in ventral view: (0) recurve absent (and with longitudinal axis): (1) recurve present; (2) recurve absent (longitudinal axis absent to support recurve). CI = 0.40, RI = 0.78.

117. Scutellum ventral surface at apex: (0) with carina extending anteriorly through V-shaped sclerite; (1) carina absent. CI = 1.00, RI = 1.00.

118. Scutellum in ventral view: (0) triangular shaped and much less than ½ the width of the scutum; (1) or not. CI = 0.50, RI = 0.0.

### Metanotum

119. Scutellum posterior margin: (0) no margin or edge; (1) slight margin or remnant edge; (2) short, round triangular projection (converging sides); (3) long triangular projection (parallel or subparallel sides for part of length); (4) short projection with truncate apex, gradually converging sides; (5) short projection with truncate apex, rapidly converging sides; (6) short projection, gradually converging sides with emarginate apex; (7) short triangular projection (converging sides) but truncate apex; (8) broad, subparallel projection with truncate apex; (9) short, sharp triangular projection (converging sides). CI = 0.64, RI = 0.80.

120. Scutellum anterior margin: (0) straight to very slightly projecting; (1) moderately projecting and broadly rounded; (2) sinuate; (3) truncate (rounded at anterio-lateral edges); (4) slightly emarginate; (5) strongly projecting and broadly rounded. CI = 0.55, RI = 0.0.

121. Alar ridge sides on scutellum excluding anterior projection towards posterior: (0) narrow in width and sides slightly converging basally; (1) wide and sides strongly converging basally; (2) wide and sides slightly converging basally. CI = 0.40, RI = 0.76.

122. Mediophragma, ventral margin cleft shape: (0) deeply emarginate (sides subparallel); (1) shallowly emarginate (sides obliquely angled). CI = 0.50, RI = 0.0.

123. Mediophragma: (0) with a distinct longitudinal ridge; (1) with a partial ridge (remnant easily visible); (2) ridge absent. CI = 0.50, RI = 0.86.

### Meso- and metaventrite and meso- and metaleg

124. Mesosternum: (0) with a transverse and longitudinal low ridge forming a cross shape of various forms; (1) with a U shaped depression intersected by a longitudinal ridge; (2) with a transverse ridge with a very slight medial expansion anteriorly; (3) transverse ridge medially narrowly smoothly expanded ventrally; (4) with a longitudinal ridge expanded anteriorly; (5) with a transverse ridge expanded slightly anteriorly at middle forming a truncate anterior margin; (6) with a transverse ridge (in *Amietina* extending anteriorly gradually towards middle). CI = 0.66, RI = 0.57.

125. Meso- and metatibia: (0) narrow and only slightly expanded apically; (1) broad and greatly expanded apically. CI = 0.25, RI = 0.25.

126. Metatarsi: length of first metatarsomere compared to second: (0) first nearly 2X or more the length of second; (1) first only slightly longer than second. CI = 0.33, RI = 0.50.

127. First metatarsomere: (0) lengthened and greatly expanded laterally; (1) neither lengthened and expanded laterally. CI = 0.50, RI = 0.50.

128. First metatarsomere: (0) distinctly fringed on both lateral edges with dense combs of setae; (1) distinct setal fringes lacking. CI = 0.33, RI = 0.0.

129. Metacoxal separation at middle: (0) relatively large, coxae distinctly separated throughout and ventrite projection anteriorly at middle separating coxae truncate at apex; (1) relatively narrow, coxae not distinctly separated anteriorly, in contact or nearly so, ventrite projection pointed at apex. CI = 0.50, RI = 0.85. Note that this character appears to be quite variable within the Eurysternini.

130. Metatibia: (0) elongate and parallel sided at middle ½; (1) or not. CI = 0.25, RI = 0.57.

131. Metasternum between mesocoxae: (0) diverging anteriorly; (1) approximately parallel sided; (2) converging anteriorly. CI = 0.22, RI = 0.58.

132. Metasternum posteriorly: (0) with a shallow depression; (1) depression absent. CI = 0.33, RI = 0.0.

133. Metendosternite basal stalk: (0) sides diverging throughout length; (1) sides parallel (sometimes slightly sigmoidal) to slightly converging . CI = 0.33, RI = 0.33.

## Results

**Figure 1.**
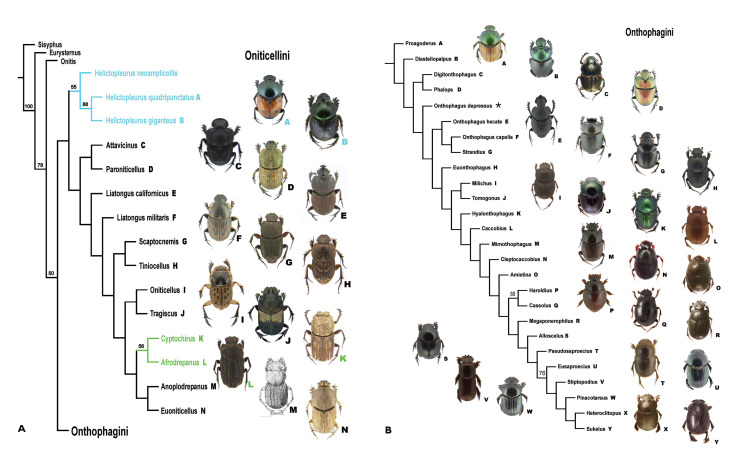
**A, B** Cladogram found using Piwe weighting with K values of 8-10. This topology is considered to be the best supported in this study. Bootstrap values above 50% found for nodes indicated. **A** Oniticellini. **B** Onthophagini. * = no taxon image.

**Figure 2.**
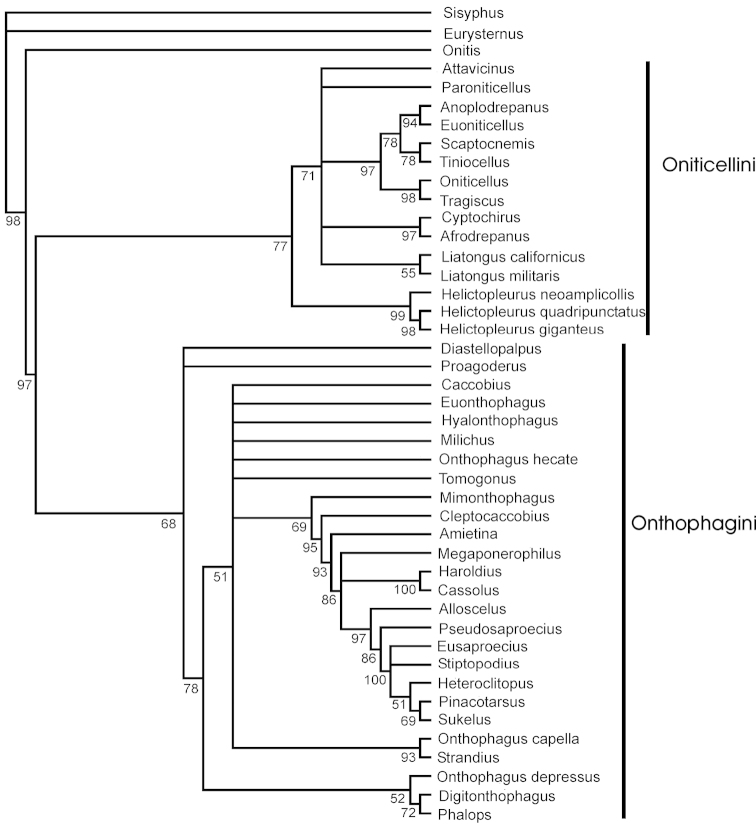
Bayesian 50% majority rule consensus tree with confidence values above 0.5 for nodes indicated.

**Figure 3.**
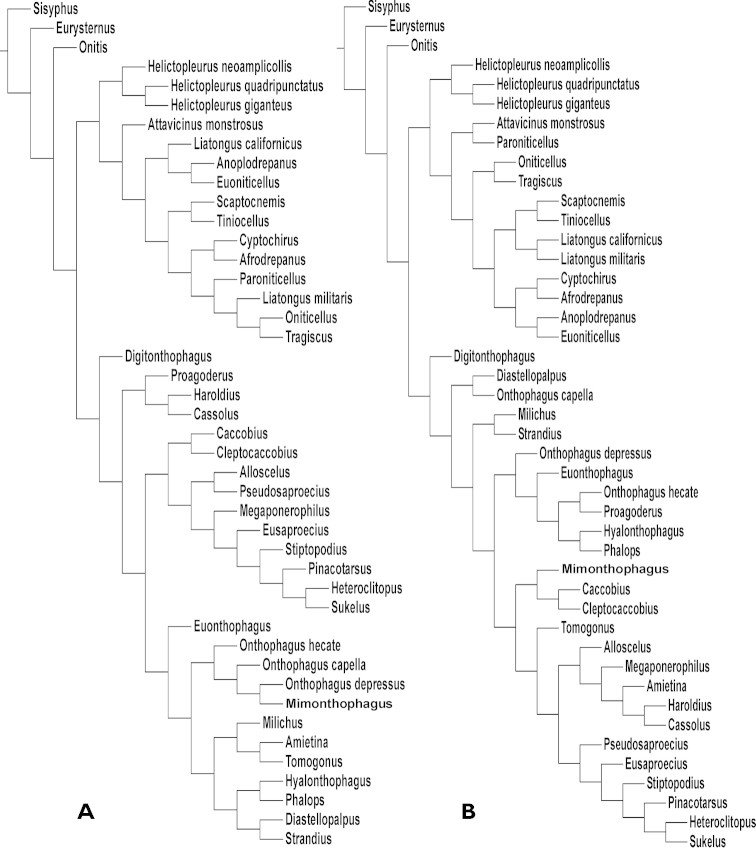
Cladograms found with extreme weighting using Piwe: **A** K value of 1 (left side) **B** K value of 3 (right side).

**Figure 4.**
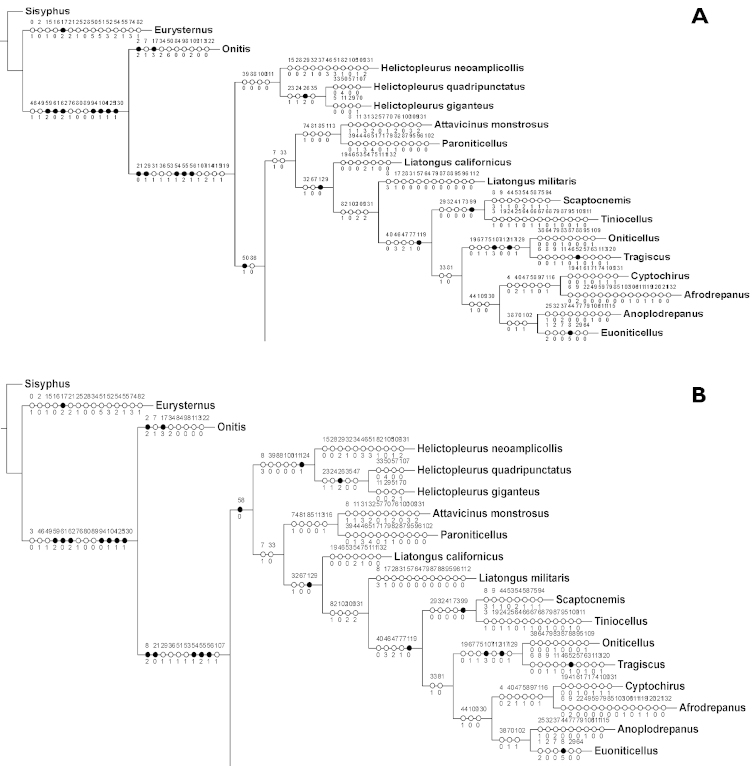
Oniticellini topology with characters and states shown. **A** Topology above using unweighted data shows the tribe as paraphyletic without the Onthophagini **B** Topology below using the weighted data with K value of 10 shows the tribe as monophyletic.

**Figure 5.**
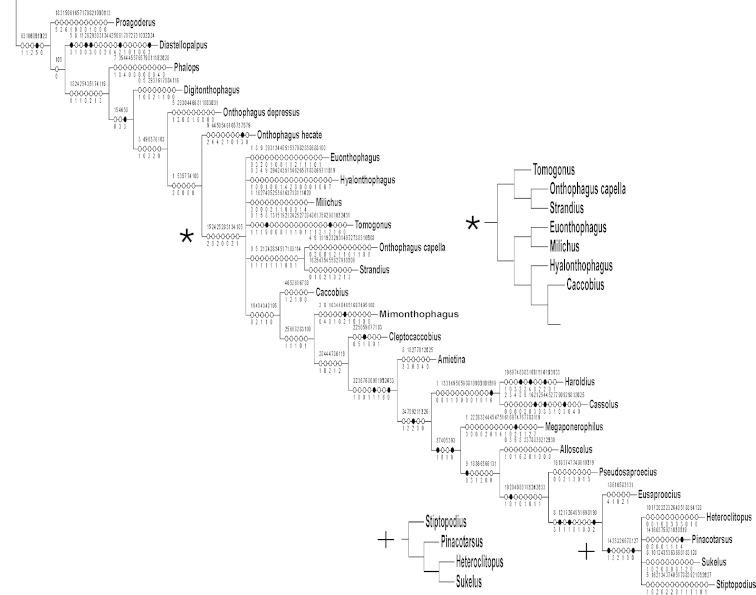
Onthophagini topology, with characters and states shown and based on unweighted data. Inserted clades are the resolved topologies found with Piwe weighting using K values of 11-30.

The hypothesis that the Oniticellini and Onthophagini constitute a monophyletic group is strongly supported by this study (Figs 1–5). No outgroup taxa were found within the ingroup in any of the analyses. Analysis of the unweighted data using parsimony produced a total of six trees (strict consensus with character support in Figs 4A, 5), each with a length of 825 steps, and overall CI and RI of 0.29 and 0.50, respectively. Alternative topologies in the step-like tree are due to two polytomies within the onthophagines, one located approximately in the middle of the topology involving alternate relationships of six taxa and the other located at the apex in four probable termite associated taxa. The Bayesian analysis shows unresolved clades near or at the base in both the Oniticellini and the Onthophagini (Fig. 2).

**Figure 6.**
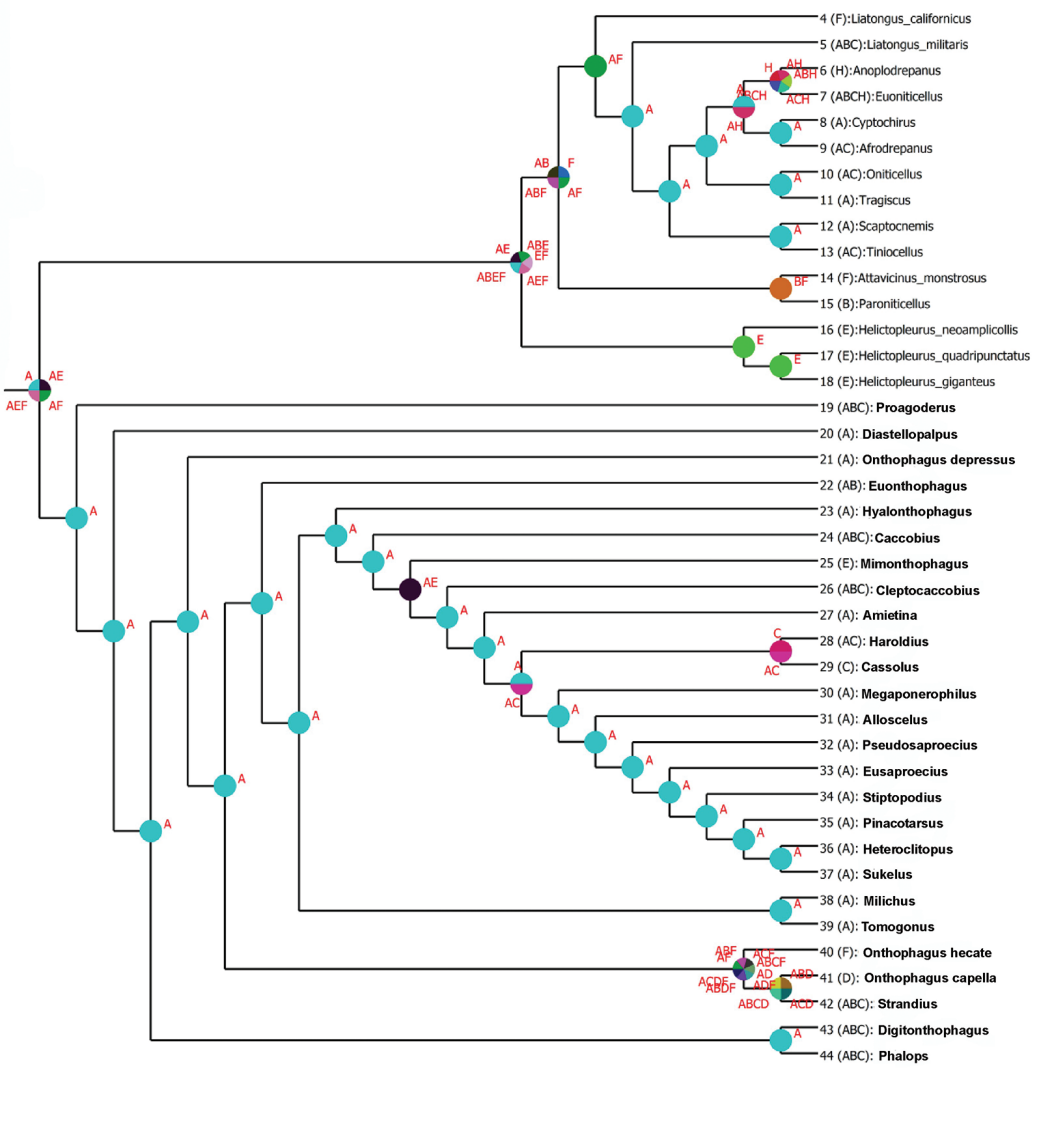
Biogeographical analysis using S-DIVA of the Oniticellini and Onthophagini showing relative probabilities of ancestral area alternative distributions. Each color and associated letter code represents a biogeographic area used in the analysis: **A** Afrotropical **B** Palaearctic **C** Oriental **D** Australasian **E** Madagascar **F** Nearctic **G** Neotropical **H** Caribbean. Font in bold indicates Onthophagini taxa.

The tribe Onthophagini is monophyletic in all analyses. For the Oniticellini two results were discovered. The tribe is monophyletic in the Bayesian tree (Fig. 2) and the Piwe weighted trees with K values from 1–10 (Figs 1A, 3, 4B). In contrast, the parsimony tree using unweighted characters and the Piwe weighted trees with relatively high K values from 11–50 show the Helictopleurina as sister to the remaining oniticellines + onthophagines (Fig. 4A). K values of 44 and higher resulted in one of the six unweighted trees found. This was an expected result since with higher K values the weighting becomes increasingly reduced and eventually becomes equivalent to unweighted data. Topologies found using K values = 11–30, are identical and similar to the unweighted data topology except that the two polytomies seen in the latter are fully resolved (Fig. 5, two small clade inserts).

K values of 8–10 also resulted in identical topologies (Fig. 1) while those of 4–7 resulted in minor rearrangements of taxa within the Onthophagini. Rearrangements occurred at the base or in more derived clades, via taxa shifting up or down one node, or by adjacent taxa becoming sisters. As expected, weighting generally increases tree length compared to unweighted data, except in cases of minimal weighting (i.e., high K values) that result in the same topology found using unweighted data. But moderate weighting of K values = 8–11 only increased the tree length by two steps (825 to 827). In contrast, K values of 1, 2, and 3 increased the tree length by 31–64 steps (lengths were 889, 874, and 856 steps respectively). Additionally, analyses with the very lowest K levels show trees the most altered (Fig. 3) compared to the unweighted analyses. Therefore the best hypothesis is considered to be the fully resolved parsimony topology using K values of 8–10 (Fig. 1) due to only moderate data weighting, the increase in tree length by only two steps, and congruence with the Bayesian analysis (Fig. 2).

### Clades within the Oniticellini

In all analyses, the Helictopleurina and Drepanocerina are monophyletic (supported by four and six controverted synapomorphies respectively) while the Oniticellina is paraphyletic. The sister relationships of *Tiniocellus + Scaptocnemis, Tragiscus + Oniticellus*, and *Anoplodrepanus + Euoniticellus* are also invariant in all analyses. *Attavicinus monstrosus* (Bates), formerly placed in *Liatongus* (see Philips and Bell 2008), does not share common ancestry with the other *Liatongus* taxa. The clade consisting of *Attavicinus* + *Paroniticellus* is maintained except in the most extreme weighting (K = 1 (and 2, not illustrated); Fig. 3). In those two topologies, *Paroniticellus* is shifted to a more derived position in the tree and *Attavicinus* is a basal lineage and sister to all remaining Oniticellina + Drepanocerina. Also, the clade composed of *Liatongus militaris* as sister to *Tragiscus* + *Oniticellus* is shifted to an apical origin. The remaining oniticellines show only minor differences in arrangement in the trees with K values < 6 compared to that seen in with K values of 8–10.

### Clades within the Onthophagini

This tribe is a well-defined monophyletic group based on both Bayesian and parsimony analyses, and using either unweighted or weighted data. In the unweighted topology, five states (one uncontroverted) support monophyly. The relationships found with K values = 8–10 are identical (Fig. 1B) and differ from those found at higher K values of 11–30 (Fig. 5) in that the basally originating *Phalops* and *Digitonthophagus* are sister taxa in the former instead of successive branches in the latter. There are also minor differences of the position of some taxa in the middle of the tree (from *Onthophagus hecate* through to *Milichus*). Trees based on stronger weighting with K = 1–7 values (see Fig. 3) place *Digitonthophagus* as basal and, in trees from K = 2–7, *Onthophagus capella* + *Diastellopalpus* as the next most apical clade. In the K = 4–7 trees, a third basal clade consisting of *Proagoderus* + *Phalops* is seen.

The trees found with the most extreme weighting (K values of 1–3) compared to all others are the most different (Fig. 3); the topology found with K = 2 (not illustrated) contains elements of both. Changes include the shifting to a more apical origin of both *Proagoderus* and *Phalops*.

The probable symphile taxa (from *Amietina* to the tree apex) with the most derived features have strong support for monophyly; all included nodes are supported by a minimum of one uncontroverted and two controverted states. These lineages are not dung feeders but necrophilous (*Amietina*), myrmecophilous (*Haroldius*, *Megaponerophilus*, *Alloscelus*, and likely *Eusaproecius* and *Pseudosaproecius*) or termitophilous (*Heteroclitopus*, *Pinacotasus*, *Stiptopodius*, and probably *Sukelus*). Inquiliny most parsimoniously evolved only once in the common ancestor of *Haroldius* through to *Sukelus*. Support for this clade is based on five character states, including one uncontroverted. The Alloscelina (*Alloscelus*, *Haroldius*, and *Megaponerophilus*) is found within this larger clade but there is no support for its monophyly in any of the tree searches performed.

## Discussion

These results and the morphological studies of Philips et al. (2004b), Edmonds and Halffter (1978) and Zunino (1983) and the molecular studies of Villalba et al. (2002), Ocampo and Hawks (2006) and Mlambo et al. (2015) support monophyly (or at least a close relationship) of each of these two tribes. In contrast, the molecular study of Wirta et al. (2008) does not support either a monophyletic Oniticellini or Onthophagini. The base of their tree contains two large clades of onthophagines, and between those lineages and the apical helictopleurines are a mix of clades composed of both the oniticellines and onthophagines. In the molecular study of Monaghan et al. (2007), the oniticellines and onthophagines were always a single clade based on parsimony or Bayesian 9 partition models. But in other tested models (Bayesian 7 partition, maximum likelihood, parsimony length invariant, and POY models), this group was paraphyletic. Additionally, the tribe Oniticellini was monophyletic in the Bayesian 7 partition analysis (their Fig. 2), but paraphyletic in the nine-partition, maximum likelihood, and three parsimony analyses via the inclusion of either two or four species of onthophagines (one parsimony tree shown in their Fig. 3). In contrast to the results here, the genus *Helictopleurus* was in a relatively derived position and hence, there is no support for a monophyletic Oniticellini without the inclusion of the helictopleurines. Lastly, the onthophagines were always polyphyletic. Similar problems of non-monophyly of these tribes are seen in Mlambo et al. (2015), using molecular evidence from four genes in a study of African taxa, where the tribe Onthophagini is paraphyletic without the Oniticellini.

In contrast to all other studies, Bai et al. (2011) found the sisyphines positioned within the oniticellines. This study relied on hind wing and body morphological characters and the result is most likely due to evolutionary convergence. Additionally, Génier (2009) moved the genus *Eurysternus* as a subtribe to the Oniticellini, but evidence herein does not support this placement.

### The Oniticellini Clades

Traditionally the Oniticellini are divided into three subtribes, the Drepanocerina, Helictopleurina, and the Oniticellina (e.g., Halffter and Matthews 1966). In this study the Drepanocerina and Helictopleurina are monophyletic. Recognition of the Helictopleurina as a tribe would eliminate possible paraphyly in the Oniticellini. This was done for perhaps the first time in Orsini et al. (2007), although their study provides little evidence for doing so with a single outgroup and only Madagascar species represented.

### Drepanocerina

The Drepanocerina currently contain 12 genera but until recently included only two, *Cyptochirus* and *Drepanocerus*. Even the genus *Cyptochirus* was previously considered a synonym of *Drepanocerus* (e.g., Arrow 1931, Halffter and Matthews 1966). Simonis and Zunino (1980) validated the genus, which at that time included several Oriental species now placed in *Sinodrepanus* (Simonis 1985a). Simonis (1985b) later hypothesized *Drepanoplatynus* and *Sinodrepanus* as sister genera and this clade in turn sister to *Drepanocerus*, relationships not supported in Roggero et al. (2015). New genera in this subtribe were recently created (Krikken 2009, Barbero et al. 2009a,b, and Roggero et al. 2015) including *Afrodrepanus* Krikken, *Clypeodrepanus* Krikken, *Drepanellus* Barbero, Palestrini and Roggero (now a junior synonym of *Latodrepanus*), *Eodrepanus* Barbero, Palestrini and Roggero, *Epidrepanus* Roggero, Barbero, and Palestrini, *Latodrepanus* Krikken, *Paraixodina* Roggero, Barbero, and Palestrini and *Tibiodrepanus* Krikken. A recent phylogenetic study of morphology by Roggero et al. (2015) including representatives of all genera has now hypothesized relationships within this subtribe.

The two representatives in this study, *Cyptochirus* and *Afrodrepanus*, were found as sister taxa. The Drepanocerina may not be monophyletic based on preliminary studies mentioned but not discussed in Barbero et al. (2009b), perhaps due to the relative heterogeneous morphologies of the genera. Krikken (2009) does give a morphological description of the group but later notes that support for their monophyly is negligible. Roggero et al. (2015) using *Anoplodrepanus* as a single outgroup gives a relatively weak test for monophyly. But based on the results in this study and the hypothesized close relationship of taxa included within the Drepanocerina, there is support for the monophyly of the clade. Notably, *Scaptocnemis* and *Anoplodrepanus* have been placed by some authors in the Drepanocerina previously, but this study supports their placement in the Oniticellina as suggested earlier by Branco (2010) and Krikken (2009). The Drepanocerina as defined herein is therefore restricted to the Afrotropical-Oriental regions.

### Helictopleurina

The Madagascar endemic helictopleurines are composed of only two genera, the speciose *Helictopleurus* with ~80 species and the monotypic *Heterosyphus*. The included species of *Helictopleurus* representing three subgenera are strongly supported as monophyletic. The rare *Heterosyphus* was not available for study. When Paulian (1975) described this genus he placed it in the Canthonini and discussed its unusual morphology, surmising that it was not closely allied with this clade. But he also stated that this taxon might be related to *Helictopleurus* based on the form of the head and legs. Similarities were noted with *Sisyphus* in the form and sculpture of the prothorax and that the presence of a visible scutellum separates it from *Onthophagus*, although this last character is present to various extent within species of *Helictopleurus* as well. Halffter and Edmonds (1982) may have been the first to place *Heterosyphus* in the Helictopleurina. The fairly well supported molecular study of Wirta et al. (2008) shows this taxon to be a derived *Helictopleurus*. Therefore, *Heterosyphus* Paulian should be considered a junior synonym of *Helictopleurus* d’Orbigny so that the species becomes *Helictopleurus sicardi* (Paulian).

The position of the Helictopleurina in the moderately or heavily weighted topologies supports the recognition of the group as either a monophyletic subtribe or tribe. In contrast, the unweighted or slightly weighted topologies support the creation of a new tribe to avoid a paraphyletic Oniticellini. But molecular evidence from Monaghan et al. (2007) in some of their analyses suggests that the helictopleurines are derived from within the oniticellines. Therefore although the results found in this study support the helictopleurines as either a subtribe or tribe, current molecular evidence suggests that this clade should continue to be recognized as a subtribe.

### Oniticellina

The subtribe Oniticellina is paraphyletic, although some generic sister relationships are invariant (see in results above). *Euoniticellus* and *Paroniticellus* have been classified as subgenera of *Oniticellus* in the past (Halffter and Matthews 1966) but neither appears phylogenetically close to *Oniticellus* and their generic status is supported. Interestingly, some evidence exists that the monotypic *Paroniticellus* and *Oniticellus*, (perhaps only the subgenus *Oniticellus, sensu stricto*) are closely related, as larvae of both are characterized by numerous setae on the head capsule (Lumaret 1979). But immatures will first need to be examined over a broader range of species to confirm this potential synapomorphy. The Jamaican endemic *Anoplodrepanus* is sister to *Euoniticellus* and also probably a valid genus.

While an African *Euoniticellus* was included in this study, the single New World species *Euoniticellus cubiensis* Laporte from the West Indies (Cuba, Jamaica, Isla de la Juventud, and the Bahamas (Woodruff 1973, M.A. Ivie pers. comm.)) was not. Zunino (1982) states that this species (referred to as *Oniticellus cubiensis*) “belongs though in a rather isolated position, to the genus *Euoniticellus*.” A more recent phylogeny of *Euoniticellus* by Cambefort (1996) solidly supports its placement in this genus.

Several genera not included also deserve brief comments. The monotypic genus *Scaptodera* was removed from generic synonymy by Paulian (1986), but might be a *Liatongus* based on its previous placement. *Ixodina* is often considered a synonym of *Drepanocerus* (e.g., Davis et al. 2008) but Krikken (2009) and Roggero et al. (2015) list this genus as valid and part of the Drepanocerina. The species now placed in the Southeast Asian *Yvescambefortius* (Ochi and Kon 1996) was originally placed in *Oniticellus* by Gillet (1926) and later in *Tiniocellus* by Janssens (1953). Ochi and Kon (1996) hypothesized a close relationship of their new genus with the east African *Scaptocnemis*, which in this phylogeny is sister to *Tiniocellus*. Hence, it may be part of the *Scaptocnemis* + *Tiniocellus* clade.

Based on this study, a solution for monophyly within the Oniticellini subtribes outside of the Helictopleurina would be to recognize all genera (including the Drepanocerina) in a single subtribe, or alternatively, the recognition of the Drepanocerina and two or more subtribes. From the Bayesian analysis and the majority of parsimony topologies found (see Figs 1A, 4B), another possibility is a subtribe including *Paroniticellus* and *Attavincinus*, while the other would include all remaining genera together with the Drepanocerina. One could also divide the Oniticellina into three or more subtribes while continuing to recognize the Drepanocerina. Based on current evidence of relationships, a possible subtribal classification recognizing hypothesized monophyletic groups is as follows:

**Drepanocerina**: *Afrodrepanus, Clypeodrepanus, Cyptochirus, Drepanocerus, Drepanoplatynus, Eodrepanus, Epidrepanus, Ixodina, Latodrepanus, Paraixodina, Sinodrepanus* and *Tibiodrepanus*.

**Helictopleurina**: *Helictopleurus* (with *Heterosyphus* a junior synonym of *Helictopleurus*).

**Liatongina subtr. n. Philips. Type genus *Liatongus* Reitter, 1893**: *Liatongus* Reitter (monophyly of the genus needs confirmation).

Diagnosis: This subtribe can be characterized by the following two characters: The mesonotal prescutum anteriorly with the transverse plate approximately ventrally directed is narrowly rounded apically and the posterior median tormal process (epitorma) of the epipharynx is smoothly and broadly tapered throughout its length. Based on a weighted topology (K=3) the three following characters also support this clade: The prothoracic apodeme has an incomplete oblique suture/carina, one or more “extra” internal sclerites are associated with the galea, and the metanotum scutellum posteriorly has a slight margin or remnant edge.

**Oniticellina**: *Anoplodrepanus, Euoniticellus, Nitiocellus, Oniticellus, ScaptocnemisScaptodera, Tiniocellus, Tragiscus* and *Yvescambefortius*.

**Attavicina subtr. n. Philips. Type genus *Attavincinus* Philips & Bell, 2008**: *Attavicinus* and *Paroniticellus*.

Diagnosis: This subtribe can be characterized by the following combination of characters: Maxilla internal accessory sclerite is curved near the proximal apex, the protibia on the proximal side has a line of setae associated with a carina, males have a longitudinal ridge on the pronotum laterally, and the mesosternal scutum is distinctly transverse in shape.

Note that based on the most heavily weighted parsimony topologies (Piwe K values = 1 and 2), *Paroniticellus* should be placed within the Oniticellina. Based on the Bayesian topology, *Paroniticellus* may need to be placed in its own or possibly one of the other subtribes, as its relationship to the other subtribes (excluding the Helictopleurina) is unclear.

### The Tribe Onthophagini

This tribe in every analysis was a well supported monophyletic group. Philips et al. (2004b) also support the monophyly of this tribe based on morphological evidence. In contrast, molecular data in Monaghan et al. (2007), Wirta et al. (2008) and Mlambo et al. (2015) generally support a poly- or paraphyletic Onthophagini and indicate that some currently recognized onthophagines may belong elsewhere.

The topologies found herein with extreme weighting K = 1–3 topologies are considered less trustworthy compared to those found with higher K values. This conclusion is also supported by the molecular studies of Monaghan et al. (2007) and Wirta et al. (2008) that do not support either a relatively derived position of *Proagoderus* and *Phalops*. Further, Tarasov and Solodovnikov (2011) also found support for a basal origin of *Proagoderus* and *Diastellopalpus* as seen in the less strongly weighted topologies. An internal classification of the onthophagines, if desired and based on the evidence herein, would likely necessitate the creation of numerous subtribes to maintain monophyly in classification.

### The Apical Clades of the Onthophagini

Branco (1989, 1990, 1991, 1992a,b, 1995) placed several of these most derived genera together in a new group based on shared morphological features, such as antennomere number. His *Pinacotarsus* group includes eight genera (taxa in bold included in this study): *Dorbignyolus*, ***Eusaproecius***, ***Heteroclitopus***, *Krikkenius*, *Pinacopodius*, ***Sukelus*** (as *Falcidius*), *Stiptocnemis*, and ***Pinacotarsus***. The *Stiptopodius* group, first defined by Boucomont (1923) and containing some of the genera in the *Pinacotarsus* group, was redefined by Branco (1995) and includes the genera *Neosaproecius*, ***Pseudosaproecius***, ***Stiptopodius***, and *Stiptotarsus*. Of the six genera included in this study, all form a monophyletic clade at the apex of the onthophagines in both the parsimony and Bayesian analyses. Branco hypothesized the *Stiptopodius* group as less derived than the *Pinacotarsus* group and this is approximately seen in Figs 1 and 2. But neither group is monophyletic in any of the topologies discovered in this study.

### The Genus *Onthophagus*

As a very preliminary test of monophyly for *Onthophagus*, three species were studied, including one Nearctic, an Australian, and one Ethiopian. Also included were four taxa that are usually considered subgenera of *Onthophagus* (i.e., *Digitonthophagus*, *Diastellopalpus*, *Proagoderus*, and *Strandius*), as well as *Euonthophagus*, *Hyalonthophagus*, and *Mimonthophagus*. All will be considered *Onthophagus, sensu lato* in the discussion below.

In this study, *Onthophagus*, in either the strict or broad sense, is not monophyletic. *Onthophagus, sensu stricto*, typically appears in three clades. *Onthophagus, sensu lato*, appear in various topologies in no fewer than four to as many as nine separate lineages. *Strandius* often appears as part of a clade of *Onthophagus* while *Euonthophagus*, *Hyalonthophagus* and *Mimonthophagus* are justified as genera evolutionarily distinct from *Onthophagus*. In regards to *Onthophagus, sensu stricto*, molecular evidence from Monaghan et al. (2007) also does not support monophyly. Tarasov and Kabakov (2010) discuss the difficulty with placing species within the subgeneric classification. Furthermore, the creation of subgenera based on Asian species that are derived from African lineages will certainly create issues of monophyly for the latter.

Two phylogenetic studies have been done that concentrate mainly on *Onthophagus*. One on the “*Serrophorus*” complex (Tarasov and Solodovnikov 2011) used morphological data and included 39 species of *Onthophagus*. This work also explored the use of male endophallic characters in the group; from 74 parsimony informative characters used, 45 (~60%) were coded from male genitalia. Emlen et al. (2005) used a sample of 48 *Onthophagus* species, employing up to seven gene sequences. Monophyly of the included taxa is assumed in both studies and it is possible that this is not the case. Regardless, more taxonomically broad studies on this genus and with molecular data are certainly needed to clarify relationships among subgenera and species groups. More genera then are currently recognized will likely be needed to delimit monophyletic clades in this speciose genus.

From various sources (Wikispecies website accessed 3 N. 2015, Zunino 1979, Lumaret and Kim 1989, Tarasov and Solodovnikov 2011, Tagliaferri et al. 2012), the subgenera of *Onthophagus* that perhaps are most commonly accepted are listed below (taxa in bold included in this study). Excluding five that most dung beetle workers recognize at the generic level (***Diastellopalpus***, ***Digitonthophagus***, ***Mimonthophagus***, ***Proagoderus*** and ***Strandius***), this leaves 25 subgenera as follows: Onthophagus (Afrostrandius), Onthophagus (Altonthophagus), Onthophagus (Amphionthophagus), Onthophagus (Bicornonthophagus), Onthophagus (Colobonthophagus), *Onthophagus* (***Euonthophagus***), Onthophagus (Exonthophagus), Onthophagus (Furconthophagus), Onthophagus (Gibbonthophagus), *Onthophagus* (***Hyalonthophagus***), Onthophagus (Indachorius), Onthophagus (Macronthophagus), Onthophagus (Matashia), Onthophagus (Micronthophagus), Onthophagus (Onthophagiellus), *Onthophagus* (***Onthophagus***), Onthophagus (Palaeonthophagus), Onthophagus (Paraphanaeomorphus), Onthophagus (Parascatonomus), Onthophagus (Parentius), Onthophagus (Pseudophanaeomorphus), Onthophagus (Relictonthophagus), Onthophagus (Serrophorus), Onthophagus (Sinonthophagus) and Onthophagus (Sunenaga). Onthophagus (Phanaeomorphus) is another group that could be recognized (Lumaret and Kim 1989), as are Onthophagus (Trichonthphagus), Onthophagus (Eremonthophagus) and *Onthophagus* (*Palaeonthophagus*) (Zunino, 1979), and Onthophagus (Pseudonthophagus) Kabakov and Shokhin 2014, and see Tagliaferri et al. (2012 for the latest added above) bringing the total number of subgenera to as many as 30 (see Tarasov and Kabakov 2010 for a review of the subgenera).

### Onthophagines?

There are a number of scarabaeine species that historically have been placed in various tribes, reflecting difficulty in their classification. This often included some of the inquilinous scarabaeines with highly modified morphologies, such as flattened leg segments and the presence of trichomes (e.g., Krell and Philips 2010). The formerly recognized Alloscelina (or Alloscelini) was once placed within the Scarabaeini, which included all the “rollers” at that time (Halffter and Matthews 1966). This group included the currently recognized onthophagine genera *Alloscelus*, *Megaponerophilus* (a former subgenus of *Caccobius*), *Haroldius* (including its junior synonyms *Ponerotrogus* Silvestri and *Afroharoldius* Janssens, both synonymized by Paulian (1985), *Formicdubius* Philips and Scholtz, synonymized by Krell and Philips (2010)) and the genus *Freyus* Balthasar (now a junior synonym of the dichotomine *Paraphytus* Harold (Halffter and Matthews 1966)).

Two problematic genera are *Haroldius* Bocomont and *Cassolus* Sharp. Hanski and Cambefort (1991) classify *Haroldius* as a Canthonini, while Philips and Scholtz (2000) placed it within the Onthophagini. *Cassolus* is listed as a canthonine in Halffter and Matthews (1966), but as an onthophagine in Hanski and Cambefort (1991). In this study, *Cassolus* and *Haroldius* appear deep within the onthophagines as sister taxa and supported by 12 character states, including two uncontroverted. Together with their sister clade, they are supported by five characters, including one that is uncontroverted. But they may indeed not be onthophagines. In Bai et al. (2011) using morphological evidence, they found *Cassolus* + *Parachorius* positioned outside the onthophagine clade. A recently described species of the Oriental canthonine *Parachorius* bridges the morphological gap between these two genera, and both form a monophyletic clade according to Tarasov and Keith (2011). At this time, the effects of morphological convergence remain unclear and evidence from molecular data is probably needed to resolve the origins of both genera and their proper tribal classification.

### Antennal Pits

In Philips et al. (2004b), the Onthophagini and Oniticellini shared what was thought to be a fairly strong and intriguing synapomorphy of antennal cavities in the first (proximal) and middle antennomeres of the club. It was also seen in the canthonine *Epirinus hilaris* Péringuey but is lacking in *Epirinus silvestris* Cambefort and seven other canthonines examined in Philips et al. (2004b). Interestingly, *Epirinus hilaris* plus three other *Epirinus* species may be sister to the sisyphines, onitines, oniticellines and onthophagines, as seen in the molecular based topology of Monaghan et al. (2007). Some onitine taxa later examined, notably *Platyonitis smeenkorum* Krikken, were also noted to share this feature, while others (e.g. *Bubas* sp., *Heteronitis* sp., and *Onitis* spp.) did not. Interestingly, this feature is also found in the dichotomines *Ontherus* Erichson, *Scatimus* and “*Trichillum*” but not in *Scatrichus* and “*Pedaridium*” (F. Génier pers. comm.). Recently, Tarasov and Solodovnikov (2011) discovered that the Onthophagus subgenus Parascatonomus has only a single cavity on the first antennomere of the club. As these cavities are absent in a reasonably broad selection of other dichotomiines and coprines (and hence it is probably not a plesiomorphic condition for dung beetles), this trait appears to have independently evolved numerous times. The function of these antennal cavities is unknown.

### Nesting behavior in the dung feeders

Ancestral oniticellines and onthophagines are presumed to have been coprophagous as all of the basal lineages are dung feeders. Tunneling behavior in this clade is very similar amongst taxa and all are classified as Pattern I nesters in Halffter and Edmonds (1982), with a few notable exceptions. An alternative nesting strategy known only within the Oniticellini is dwelling or endocoprid behavior (classified as Pattern VII nesters), which can include nests within dung or nests in pits directly below the dung. Known only in *Oniticellus* and *Tragiscus* (Davis, 1989), it may also occur in *Paroniticellus* although this is currently unclear (see Halffter and Matthews 1966 and Philips and Bell 2008 for comments). Excluding *Paroniticellus*, this behavior only evolved once in the common ancestor of the sister genera *Oniticellus* and *Tragiscus*.

Both tribes are characterized typically and most distinctively by the absence of a brood ball and larvae are supplied with a brood mass that is either modified to some degree or not. Additionally, male-female cooperation is absent or limited and brood care is not known to exist. These are simple behaviors in what one could argue is a derived lineage compared to some of the more ancient origin tunneling clades where complex behaviors evolved. For example, coprines construct brood ovoids, and have extensive male-female cooperation, and brood care. But simple nesting behavior appears to be a very successful strategy evolutionary; the oniticellines and onthophagines are some of the most successful dung beetles in many ecosystems in terms of number of species, and often in terms of abundance and biomass (Davis et al. 2008).

### Inquiliny

The African endemic *Stiptopodius* and *Pinacotarsus* groups of onthophagines form a monophyletic clade as discussed above. Based on the results, all included species are probably either myrmecophilous or termitophilous. As evidenced by the relatively high diversity within this lineage, the association with social insects may be relatively ancient. In contrast, only the New World oniticelline, *Attavicinus montrosus*, is an inquiline. This species is associated with leaf-cutter ant debris piles and has a very restricted distribution in central Mexico (Philips and Bell 2008). There is no strong evidence for the age of these associations at this time.

Krikken (1982) stated that the onthophagine genera *Dorbignyolus*, *Krikkenius* and *Pinacopodius* are all termitophilous. It is also thought that several others, including *Pinacotarsus*, *Heteroclitopus*, *Stiptocnemis* and *Sukelus*, are also known or likely termitophilous (Branco 1991, 1992a,b). Directly above this termitophilous clade in a less derived position are two taxa in the genera *Eusaproecius* and *Pseudosaproecius*. *Pseudosaproecius* is considered a generalist in regards to food source by Branco (1995), with two species collected on fish carrion and a third on human dung (and see Cambefort 1984). But he also notes that nine species have been collected only at light. Similarly, only a single record from dung of a *Eusaproecius* species (of seven described) is known (Branco 1992a). The isolated dung and carrion records are probably not typical, but more likely may be supplemental food sources. A total of 26 specimens of *Neosaproecius trituberculatus* (Frey) were only collected in flight intercept traps and never at any bait in Ghana (Philips, unpublished data). Hence, it seems probable based on common ancestry, all of the species in this clade are symphiles of some type.

Above these clades (in less derived positions) in successive steps are three additional lineages also associated with social insects. The first, *Alloscelus*, is thought to be closely related to *Pseudosaproecius* (due to similar male genitalia and form of sexual dimorphism) but they do not form a monophyletic clade. The second and third, *Megaponerophilus* and *Haroldius* + *Cassolus*, are mainly myrmecophiles, with the exception of *Cassolus* where only one species of nine is known to be an ant associate (Halffter and Matthews 1966).

In summary, the evidence in this analysis suggests that there may be a single origin of inquiliny in the onthophagines. Several genera with unknown habits are probably associated with either ants (*Eusaproecius* and *Pseudosaproecius*) or termites (*Sukelus*). The other four genera may also be considered as part of this clade although future studies may shift *Haroldius* and *Cassolus* out of the onthophagines. The evolution of myrmecophily preadapting the more derived taxa for an association with the Isoptera also seems plausible.

### Necrophagy, mycetophagy, and frugivory

Necrophagy and mycetophagy are rare within the oniticellines and onthophagines and perhaps are recently evolved behaviors with several independent origins. For example, *Liatongus rhinocerulus* (Morón 1984) and some species of *Onthophagus* are specialists on basidiomycete mushroom fruiting bodies, although *Liatongus rhinocerulus* has also been found on carrion and most of the *Onthophagus* are probably generalist feeders (Halffter and Matthews 1966). Carrion feeding is a specialty of species of *Amietina*, is known in *Caccobius*, and in New and Old World species of *Onthophagus*. There are many taxa associated with feeding on millipede carrion as well, particularly in Africa, perhaps indicating a more ancient origin of this habit. Other alternative feeding habits, such as fruit feeding, may be more recently derived. One should note that the use of any of these foods by adults does not necessarily indicate their use as larval brood food.

### Biogeography

The Oniticellini and Onthophagini are postulated to be a relatively modern aged group of dung beetles by Cambefort (1991). Davis et al. (2002) noted that the antiquity of generic distribution patterns lent support for an old age of the entire tribal and subtribal lineage divergence within the Scarabaeinae. Estimates for an origin of the common ancestor of these two tribes are the late Mesozoic (Davis et al. 2008, Philips 2011). More precisely, Mlambo et al. (2015) place the origin at ~early Miocene and the Oniticellini at mid to late Miocene. An Afrotropical origin of the common ancestor of both tribes is most likely, based on this study as well as Philips et al. (2004b) and Monaghan et al. (2007). Based on the results herein, most of the lineages are restricted to the Afrotropical region (Fig. 6). While all of the early branching as well as most of the generic level lineages of onthophagines are restricted to the Afrotropics with dispersals to other regions found in typically more derived lineages, the oniticellines show a pattern of relatively ancient lineage shifts into Madagascar, Palaearctic, and Nearctic regions and, more recently, into the Orient and the Caribbean.

Another general trend of the Oniticellini appears to be successive invasions from the Ethiopian into the Palaearctic through to the Oriental region (including the Lesser Sunda Islands and Sulawesi) in many groups (e.g., *Tibiodrepanus* (Barbero et al. 2011)). Currently the Drepanocerina have eight genera endemic to Africa while the Oniticellina have two. Hence, 14 generic level clades that evolved in Africa have dispersed to other geographic regions. In the Palearctic and the Caribbean, the Oniticellina have a single endemic genus in each region (*Paroniticellus* and *Anoplodrepanus*, respectively), while the Drepanocerina have a single endemic (*Sinodrepanus*) in Southeast Asia and southern China (Davis et al. 2002). The Oniticellina are known from the Mediterranean region but, in contrast, no Drepanocerina are currently found there, although fossil deposits from England dated at 70–100,000 years BP indicate they were once present (Krikken 2009, Barbero et al. 2009b).

Based on basal branching, the oniticelline ancestors that led to the Madagascan helicopleurines, the Palaearctic *Paroniticellus*, and the New World *Attavicinus* and *Liatongus* may be relatively old dispersal events. Dispersal of an ancestral oniticelline from Africa to Madagascar during the early to middle Cenozoic may be the most likely hypothesis for the presence of the helictopleurines (see Yoder and Nowak 2006) and the clade origin has been dated to between 34 and 21 Mya (Wirta et al. 2008). In contrast and based on the low diversity of the Madagascar onthophagines, their presence there must be due to one or more relatively recent dispersal events, not including the modern accidental introductions.

The New World oniticellines belonging to *Attavicinus* and *Liatongus* may each represent separate invasions by ancestral species from Asia to North America via Beringia. The land connection between these continents has been present from the mid-Cretaceous up through the late Pliocene 3.5 Mya and several more times during the Pleistocene (Sanmartín et al. 2001). Cambefort (1991) suggests the colonization of North America by these ancestors is not older than the Pliocene. Based on comparison with probable dispersal events in natricine snakes (Guo et al. 2012), invasions may have occurred even earlier during the late Oligocene or early Miocene ~27 Mya. If dispersal via tropical forest was required, the most likely dispersal event occurred during the Eocene (~50-35 Mya), when the northern hemisphere was much warmer and humid than it is today and a continguous boreotropical forest linked the two continents. At the end of the Eocene, a mixed deciduous hardwood forest changed to conifers and the rise of the Rocky Mountains and Sierra Madre Occidental created a barrier between the western and eastern Nearctic. It is not yet clear if the restricted distribution of these taxa in western North America is due to a mountain high elevation barrier or some other cause.

The Mexican *Attavicinus monstrosus* generally appears as sister to *Paroniticellus*, a monotypic genus known from middle Asia and Turkey. This close relationship is difficult to explain compared to the latter’s alternate position in the K = 1 or 2 extreme weighted trees, where it is deep within a paraphyletic Oniticellina. But links between the eastern/western Palearctic with western Nearctic are certainly known (Sanmartín et al. 2001) and therefore a sister relationship is plausible. This lineage is sister to all other Oniticellina and Drepanocerina included in the study, evidence for its antiquity. But no doubt further support is needed for broad acceptance.

The two Nearctic *Liatongus* species, *Liatongus californicus* (Oregon and northern California) and *Liatongus rhinocerulus* (northern and central Mexico) most likely share a most recent common ancestor based on very similar external morphologies and close but disjunct distributions. The one representative included in this study, *Liatongus californicus*, nearly always appears as sister to a larger clade that has as its basal lineage an Old World species of *Liatongus*. Zunino (1982), states that these two North American species of *Liatongus* belong to the Southeast Asian *Liatongus phanaeoides* group. Thus, the position of the two included species of this genus in this study (one New World and one Old World) as sister taxa in the K = 3 weighted tree may indicate an accurate evolutionary relationship.

The Jamaican endemic *Anoplodrepanus* and an African representative of *Euoniticellus* appear as sister taxa in all topologies discovered. It is a relatively derived lineage appearing at the apex of the Oniticellini topology with its sister clade of *Cyptochirus* + *Afrodrepanus*. *Euoniticellus* has an African origin with Palaearctic and Asian species and a single New World member, *Euoniticellus cubiensis* (Cambefort, 1996). A link between the faunas of Africa and the Caribbean has been reported elsewhere for many groups of insects and studies give both vicariance and dispersal hypotheses (e.g., Brown 1978, Flint 1977, Liebherr 1988). Both *Anoplodrepanus* and *Euoniticellus cubiensis* may represent two invasions of the New World; one by perhaps by a *Euoniticellus* ancestral species that reached Jamaica and evolved into what we now recognize as *Anoplodrepanus*. A second invasion of a *Euoniticellus* ancestral species is supported by the position of *Euoniticellus cubiensis* in a phylogeny of the genus done by Cambefort (1996). From a study on lizards (Gamble et al. 2011), it is possible that these two dung beetle dispersal events happened sometime between 6.0 to 21.9 Mya (but see Wiens et al. 2009 and references within for some contrasting evidence on colonization age and diversification in frogs).

Few biogeographic studies have been completed on individual genera. But in one on *Eodrepanus* (Barbero et al. 2009b), the oldest lineages are west and central or perhaps east African and a major split between the African and Oriental clades is seen using a variety of analyses (Ochiai similarity matrix, parsimony analysis of endemicity, and dispersal vicariance analysis). Evidence also suggests the effects of an initial African-Palearctic split and later further vicariant events and at least one more dispersal event in Asia.

The Onthophagini dispersal pattern is similar to that seen in the Oniticellini, but continued into Australia and South America, as well as everywhere else where one finds dung beetles. The African, Asian, and Palaearctic regions contain a total of 30, 11, and 3 genera, respectively. But of the total 35 Onthophagini genera, most are endemic to either the African (22 genera) or Asian (4 genera) regions (Davis et al. 2002).

*Onthophagus* alone is the most widespread as well as the most speciose genus, comprising at least 45% of the species of Scarabaeinae. It is also the only onthophagine genus that has spread outside of the Ethiopian, Oriental, or Palaearctic regions. Based on Emlen et al. (2005), this group has its oldest lineages in Africa and its youngest lineages in Australia and the New World. Dispersal to the New World may have occurred earlier than in the Oniticellini before the rise of the Western Cordillera by the late Eocene, although extensive erosion largely eliminated this barrier by 30 Mya, allowing dispersal between the western and eastern Neactic (Sanmartín et al. 2001). In the New World, the Oniticellini include six species, while the Onthophagini have 139 species (Davis et al. 2002, Delgado et al. 2006). Moreover, *Onthophagus* spread into South America (95 species) and occur at least as far south as 40th parallel (Cambefort 1991), whereas the oniticellines are completely absent from this region. The high diversity of the onthophagines (compared to the oniticellines) may be due to earlier dispersal and longer time for speciation or perhaps other factors not yet clear.

## Conclusions

This study is the first with a broad range of taxa from these two tribes that strongly supports the monophyly of the Oniticellini + Onthophagini, and the Onthophagini. The Oniticellini are also monophyletic in the Bayesian analysis (Fig. 2) and in all weighted analyses with K values of 10 or less (Figs 1A, 3). In contrast, the unweighted and weighted analyses with K values of 11–30, the tribe Oniticellini is paraphyletic without the onthophagines (Fig. 4A). This is considered a less plausible hypothesis based on the lack of congruence with trees supported with molecular data (Monaghan et al. 2007, Wirta et al. 2008, Mlambo et al. 2015).

In all topologies, the Helictopleurina are monophyletic. Recognition of this clade as a tribe would eliminate the potential paraphyly of the oniticellines in unweighted and weighted topologies (K = 11–30). But, since molecular data shows the helictopleurines as more derived oniticellines and not a basally originating lineage, the group should continue to be recognized as a subtribe. The Drepanocerina is supported as monophyletic. The Oniticellina is paraphyletic without the Drepanocerina and therefore redefinition of the former and the recognition of additional subtribes is needed if one desires to maintain monophyly in the classification.

The three most thorough molecular studies on the Oniticellini and Onthophagini generally support the monophyly of the two tribes combined and the Oniticellini (Monaghan et al. 2007, Wirta et al. 2008, Mlambo et al. 2015). But in contrast to the results herein, the Onthophagini are paraphyletic without the Oniticellini. The lack of congruence and the odd placements of taxa in the two older studies reduces the confidence in these hypotheses. In Monaghan et al. (2007) and Wirta et al. (2008) the Helictopleurina are placed in a more apical position within the oniticellines in contrast to a basal position in the present results. Additionally in some analyses done in Monaghan et al. (2007), some of the onthophagines such as *Digitonthophagus* + *Phalops* are sister to the onitines + oniticellines and remaining onthophagines. There are also genera, such as *Oniticellus* and *Tiniocellus*, where species of each appear in separate clades. In Wirta et al. (2008), there is a mix of oniticelline and onthophagine taxa in the topology, as well as a species of Onitini positioned within this clade. In contrast, Mlambo et al. (2015) support the onitines as sister to the onthophagines + oniticellines as hypothesized by Philips (2004b). Lastly, the speciose genus *Onthophagus*, based on both molecular and morphological evidence, is not monophyletic and will no doubt need future subdivions. Clearly the assumption of monophyly of this genus or any of the species or generic groups proposed should not be assumed.

Certainly larger molecular data sets and perhaps large numbers of morphological characters from a broad range of taxa is needed to help stabilize our conclusions and understanding of the evolution of these two major tribes of Scarabaeinae. Morphological phylogenies have the advantage that rarely collected taxa can be included. Regardless, future molecular studies using new techniques will produce large amounts of data and will clarify the evolution of these tribes and major clade divergence times (but see Mlambo et al. 2015 for divergence estimates of Afrotropical lineages). Although further study is needed on these tribes and scarabaeine evolution in general, the picture of their extensive diversification is slowly becoming clearer.
